# Long-term effect of epigenetic modification in plant–microbe interactions: modification of DNA methylation induced by plant growth-promoting bacteria mediates promotion process

**DOI:** 10.1186/s40168-022-01236-9

**Published:** 2022-02-24

**Authors:** Chen Chen, Miao Wang, Jingzhi Zhu, Yongwei Tang, Hanchao Zhang, Qiming Zhao, Minyu Jing, Yahua Chen, Xihui Xu, Jiandong Jiang, Zhenguo Shen

**Affiliations:** 1 College of Life Sciences, Nanjing Agricultural University, Nanjing, People’s Republic of China; 2 Jiangsu Collaborative Innovation Center for Solid Organic Waste Resource Utilization, Nanjing Agricultural University, Nanjing, 210095 People’s Republic of China

**Keywords:** DNA methylation, Epigenetic modification, Microbiome engineering, Microbiome–plant interaction, Plant growth-promoting bacteria, Rhizosphere microbiome

## Abstract

**Background:**

Soil microbiomes are considered a cornerstone of the next green revolution, and plant growth-promoting bacteria (PGPB) are critical for microbiome engineering. However, taking plant-beneficial microorganisms from discovery to agricultural application remains challenging, as the mechanisms underlying the interactions between beneficial strains and plants in native soils are still largely unknown. Increasing numbers of studies have indicated that strains introduced to manipulate microbiomes are usually eliminated in soils, while others have reported that application of PGPB as inocula significantly improves plant growth. This contradiction suggests the need for a deeper understanding of the mechanisms underlying microbe-induced growth promotion.

**Results:**

We showed PGPB-induced long-term plant growth promotion after elimination of the PGPB inoculum in soils and explored the three-way interactions among the exogenous inoculum, indigenous microbiome, and plant, which were key elements of the plant growth-promoting process. We found the rhizosphere microbiome assembly was mainly driven by plant development and root recruitments greatly attenuated the influence of inocula on the rhizosphere microbiome. Neither changes in the rhizosphere microbiome nor colonization of inocula in roots was necessary for plant growth promotion. In roots, modification of DNA methylation in response to inoculation affects gene expression related to PGPB-induced growth promotion, and disruptions of the inoculation-induced DNA methylation patterns greatly weakened the plant growth promotion. Together, our results showed PGPB-induced DNA methylation modifications in roots mediated the promotion process and these modifications remained functional after elimination of the inoculum from the microbiome.

**Conclusion:**

This study suggests a new mechanism in which PGPB affect DNA methylation in roots to promote plant growth, which provides important insights into microbiome–plant interactions and offers new strategies for plant microbiome engineering beyond the perspective of maintaining inoculum persistence in soils.

Video abstract

**Graphical abstract:**

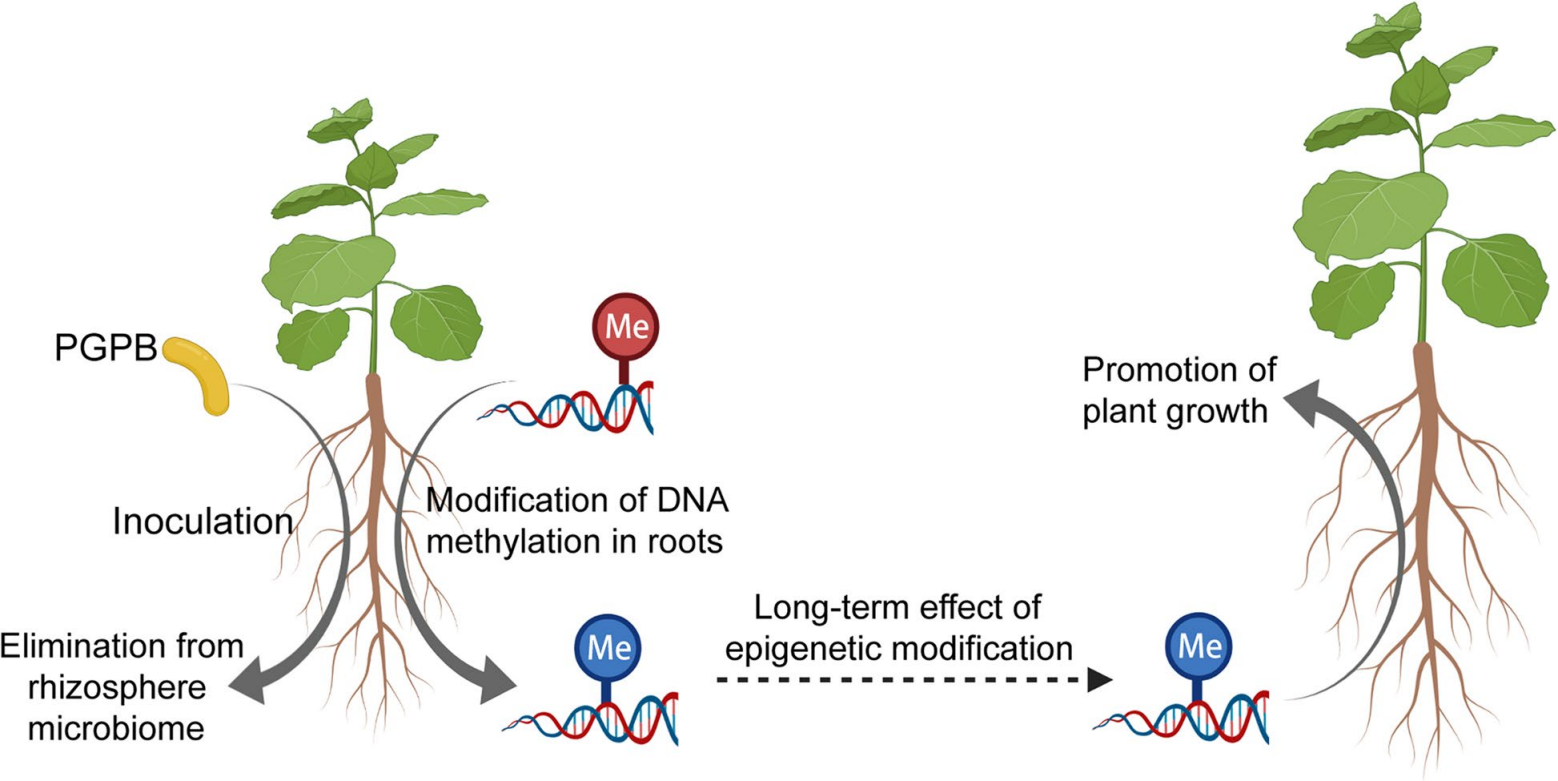

**Supplementary Information:**

The online version contains supplementary material available at 10.1186/s40168-022-01236-9.

## Introduction

In recent decades, host-associated microbial consortia have been one of the most profound discoveries with overwhelming importance for hosts, such as microbes growing in the gut or rhizosphere [1–4]. Soil microbiomes have been discussed as a cornerstone of the next green revolution, as plant-beneficial rhizobacteria may not only reduce dependence on environmentally unfriendly pesticides and agrochemicals, but also increase crop productivity, enabling more sustainable agriculture [5–8]. Among soil microbes, plant growth-promoting bacteria (PGPB) are key to engineering microbiomes for application in agriculture, including improving biocontrol and plant growth [7]. Although the mechanism underlying PGPB-mediated plant growth promotion has not been completely documented, many studies have shown that PGPB can directly facilitate nutrient acquisition or modulate phytohormone levels in plants or indirectly affect plant growth by suppressing various pathogens and enhancing the immune system or improving resistance to environmental stresses [9].

Reports of PGPB have been increasing exponentially, providing PGPB inocula for manipulating microbiomes in natural fields. However, it is usually difficult for these inocula to survive and persist in exogenous niches due to competition from indigenous microbes and/or local soil conditions, both of which might inhibit or even eliminate the inocula in the indigenous microbial consortium [6]. Indeed, in many studies, the inocula failed to flourish and their abundances decreased significantly after inoculation in exogenous soils [10–14]. Although maintaining persistence and/or sufficient abundance of inoculum for plant growth-promoting activity is not guaranteed, many studies have reported that application of PGPB as inocula significantly improved growth of different plants [6]. The paradox of continued growth promotion after the reduction or elimination of inocula in soils raises the question of what is maintained to promote plant growth and suggests the need for a deeper understanding of the mechanisms underlying PGPB-mediated plant growth promotion.

As the three-way interactions among the exogenous inoculum, indigenous microbiome, and plant are key elements of the onset and persistence of the plant growth-promoting process, we hypothesized that the following two types of variation might function continually in this process: changes in the rhizosphere microbiome and/or epigenetic changes in plants induced by the inoculum. Introduction of bacteria into soil modifies the bacterial community composition and structure, at least in the initial phase [12, 15–17]. Therefore, the altered bacterial community, including many uncultured and unknown strains, might be involved in the growth-promoting process. In plants, epigenetic modifications play an important role in regulating gene expression and coping with biotic and abiotic stresses during growth and development [18–25]. Some stress-induced epigenetic modifications may be stable and heritable, becoming epigenetic memories in the process of plant adaptation to stresses [26, 27]. To date, however, few studies have demonstrated the dynamic epigenetic modifications in plants responding to growth-promoting activities [28, 29]; thus, the role of epigenetic modification in PGPB-mediated plant growth promotion remains unknown.

DNA methylation, a common epigenetic regulation, mainly occurs at stable and easily accessible cytosine residues. Changes in DNA methylation in response to stimuli can be durable, and even potentially be passed on to subsequent generations [30, 31]. In plants, cytosine methylation occurs in three distinct sequence contexts: cytosine-guanine (CG), cytosine-H-guanine (CHG), and cytosine-H-H (CHH), where H indicates adenine (A), cytosine (C), or thymine (T). Mechanisms involved in DNA methylation maintenance are well documented [32–35]. Cytosine modifications in the CG context are maintained by methyltransferase 1, those in the CHG context are maintained by chromomethylase 2 (CMT2) or 3, and those in the CHH context are maintained by domains rearranged methylase 2 or CMT2. DNA demethylation is catalyzed by a family of DNA glycosylases, including repressor of silencing 1, demeter, and demeter-like 2 and 3. Despite the enormous progress made in understanding the molecular mechanisms regulating DNA methylation in response to environmental stimuli, most studies have used model plants, particularly *Arabidopsis*, and thus how DNA methylation mediates the stress responses of native plants in their natural habitats is largely unknown [27].

Pokeweed (*Phytolacca americana* L.), a native perennial herb in North America, is able to flourish in many habitats and has become a common invasive alien in China. *P. americana* is a Mn/Cd hyperaccumulator which has great potential for bioremediation of heavy metal (HM)-contaminated soils. To facilitate the use of a HM hyperaccumulator in bioremediation, the strategy of microbe-assisted phytoremediation has been suggested [36]. Here, we isolated two PGPB, *Bacillus* sp. PGP5 (Supplementary Materials) and *Arthrobacter* sp. PGP41 [36], to better implement PGPB-assisted phytoremediation of HM-contaminated environments by *P. americana*. Our previous study showed genetic variation is not likely to be responsible for the wide ecological distribution of *P. americana* while phenotypic plasticity plays a major role in its responding to different environments [37]. As phenotypic plasticity can be mediated through epigenetic regulation [38, 39], epigenetic states may be altered when *P. americana* is exposed to external stimuli, such as stimulus from PGPB, thereby ultimately affecting plant growth. In this study, we investigated the influence of PGPB inocula on rhizosphere microbiome assembly during plant development, the taxonomic and functional differences between inoculated and non-inoculated rhizosphere microbiomes, and the role of DNA methylation in PGPB-mediated plant growth promotion. Inoculation with either strain significantly increased the growth of *P. americana*. The successional dynamics of the rhizosphere microbiome after inoculation were analyzed at both the taxonomic and functional levels by amplicon or metagenomic sequencing. Meanwhile, changes in gene expression and DNA methylation in *P*. *americana* in response to PGP5 and PGP41 inoculation were assessed by transcriptome sequencing and whole-genome bisulfite sequencing (WGBS, a whole-genome single-base resolution analysis of DNA methylation), respectively (Fig. S1). The plants were treated with zebularine (Zeb), a DNA methylation inhibitor, to further investigate the role of DNA methylation in the growth-promoting process; meanwhile, sterilized soil was used to investigate the role of the rhizosphere microbiome (Fig. S1). A model of a two-step process of interactions between the microbiome and plant was proposed; the model comprehensively documents the dynamic regulation processes of both the rhizosphere microbiome and plant. Our results provide the first evidence for the mechanism through which PGPB affect DNA methylation to promote plant growth as well as important insights into microbiome engineering, beyond the perspective of maintaining inoculum persistence in soils.

## Results

### Rhizosphere microbiome assembly is mainly driven by plant development

**Fig. 1:**
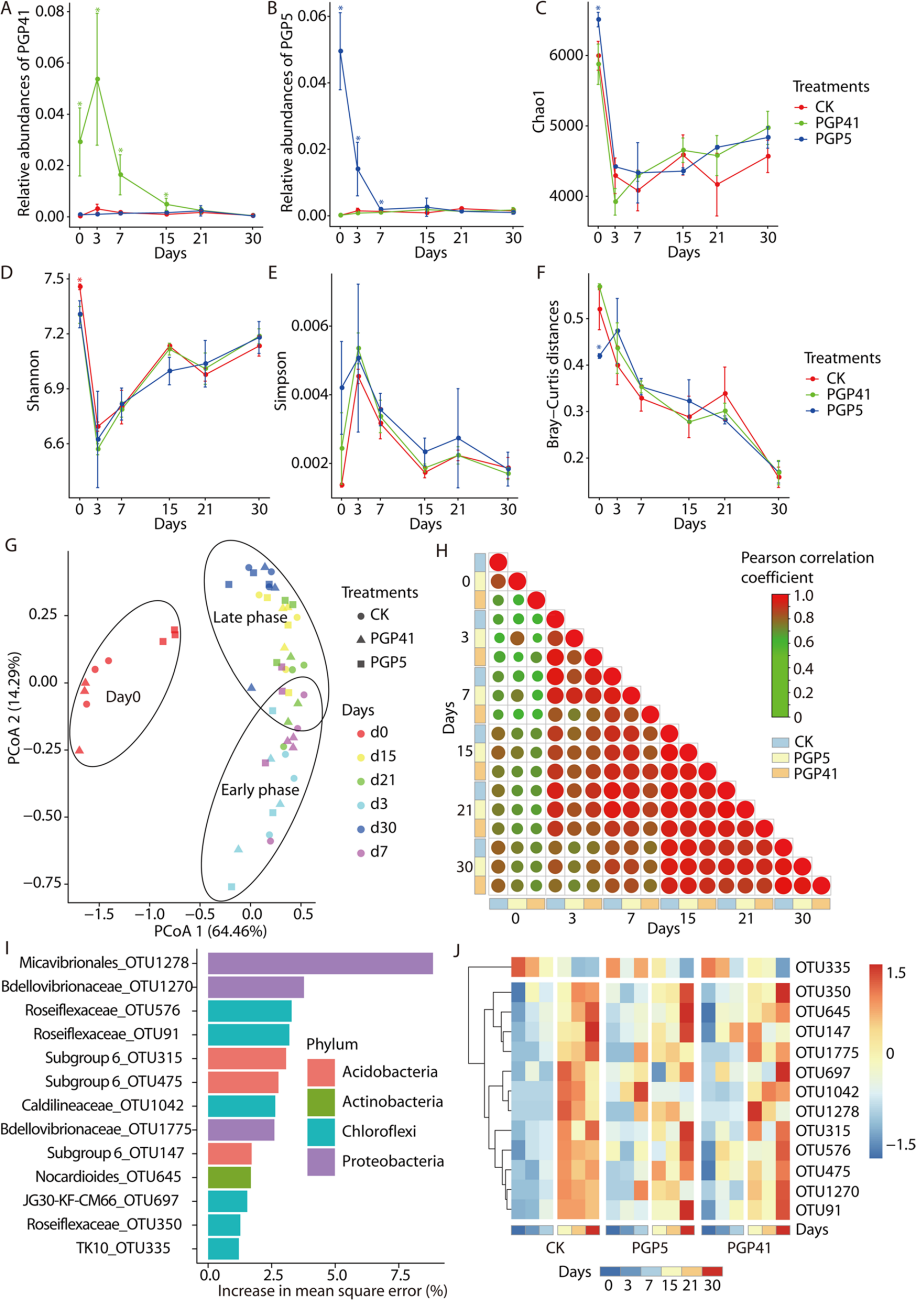
Taxonomic variation in the rhizosphere microbiome induced by roots. **A**, **B** The relative abundances of strains PGP41 (**A**) and PGP5 (**B**) in the rhizosphere microbiome-based OTUs that 100% matching to 16s rRNA gene sequences of the strains (GenBank accession number: MG839712 and MH087460 respectively). **C**–**E** Changes in α-diversity indices, including Chao1 (**C**), Shannon (**D**), and Simpson (**E**) indices. **F** Bray–Curtis distances between the microbiomes of the last samples collected (30 days) and samples from each time point (0–21 days) decreased with plant residence and development. Asterisks indicate significant differences (Duncan’s test, *P* < 0.05). **G** PCoA showing the microbiome shift with plant residence and development. **H** Pairwise correlations between samples showed similar trends of variation in inoculated and control microbiomes, and the rhizosphere microbiome became stable after 15 days of transplantation. Negative and positive correlations are displayed in green and red colors, respectively. The circle size and color intensity stand for correlation coefficients. **I** The bacterial biomarkers identified by random forests regression of their relative abundances in CK against plant residence time. **J** Heatmap based on relative abundances of biomarkers in CK and inoculated (PGP5 and PGP41) soils showing similar variation trends along with plant residence time in different soils

To explore the effects of PGPB and plant development on microbiome assembly, we investigated changes in the rhizosphere microbiome over a period of 30 days after inoculation and plant cultivation treatments. High abundances of both inocula (strains PGP5 and PGP41) were detected on day 3, followed by a rapid decrease to the same level in control soils, indicating that the inocula failed to thrive in soils (Fig. 1A, B). Measures of α-diversity revealed variation in diversity over time during plant development (Fig. 1C–E). The original bacterial community (day 0) had the highest α-diversity, while the day 3 community had the lowest. From day 3 to day 30, the α-diversity of all communities increased with time. No significant change was detected between samples treated only with plant cultivation treatments (CK samples) and samples treated with a combination of plant cultivation treatments and inoculation with strain PGP5 (PGP5 samples) or strain PGP41 (PGP41 samples). The variation in α-diversity during the late phase (15–30 days) was more moderate compared to that in the early phase (0–7 days), indicating that the rhizosphere microbiome gradually became stable. Similar results were also revealed by β-diversity analyses (Fig. 1F–H). In principal coordinate analyses (PCoA) of Bray–Curtis distances of all soil samples, day 0 samples clustered together, far from the samples with plant residence (CK, PGP5, and PGP41 at different times) on the first coordinate axis (Fig. 1G). For soil samples with plant residence, the late phase samples shifted far from the early phase samples across plant developmental stages on the second coordinate axis, with overlaps on the edge between early and late phases (Fig. 1G). No separation of soil samples with different treatments was detected, indicating consistent trends of plant development-dependent shifts of the rhizosphere microbiome among treatments. In addition, the Bray–Curtis distance between rhizosphere microbiomes collected at the end (day 30) and those collected at each time point decreased with increasing plant residence time for all treatments (Fig. 1F). Furthermore, pairwise correlation analyses revealed that the rhizosphere microbiome varied dramatically at day 3, then gradually stabilized after 15 days of root development regardless of treatments, with or without inoculation (Fig. 1H). We further identified operational taxonomic units (OTUs) correlated with root residence time using a random forests model, and 13 OTUs were detected (Fig. 1I). Although the relative abundances were low in the early phase, most of the OTUs remained at high levels during the late phase (Fig. 1J). The pattern of higher levels in the late phase compared to the early phase was also detected in the samples with inoculation treatments (Fig. 1J). Together, these results indicate that the rhizosphere compartments might have a recruitment effect on the rhizosphere microbiome, which stabilized the rhizosphere microbiome after forming intimate relationships between roots and specific bacteria.

### Variations in the rhizosphere microbiome induced by inocula are limited to the early phase

**Fig. 2:**
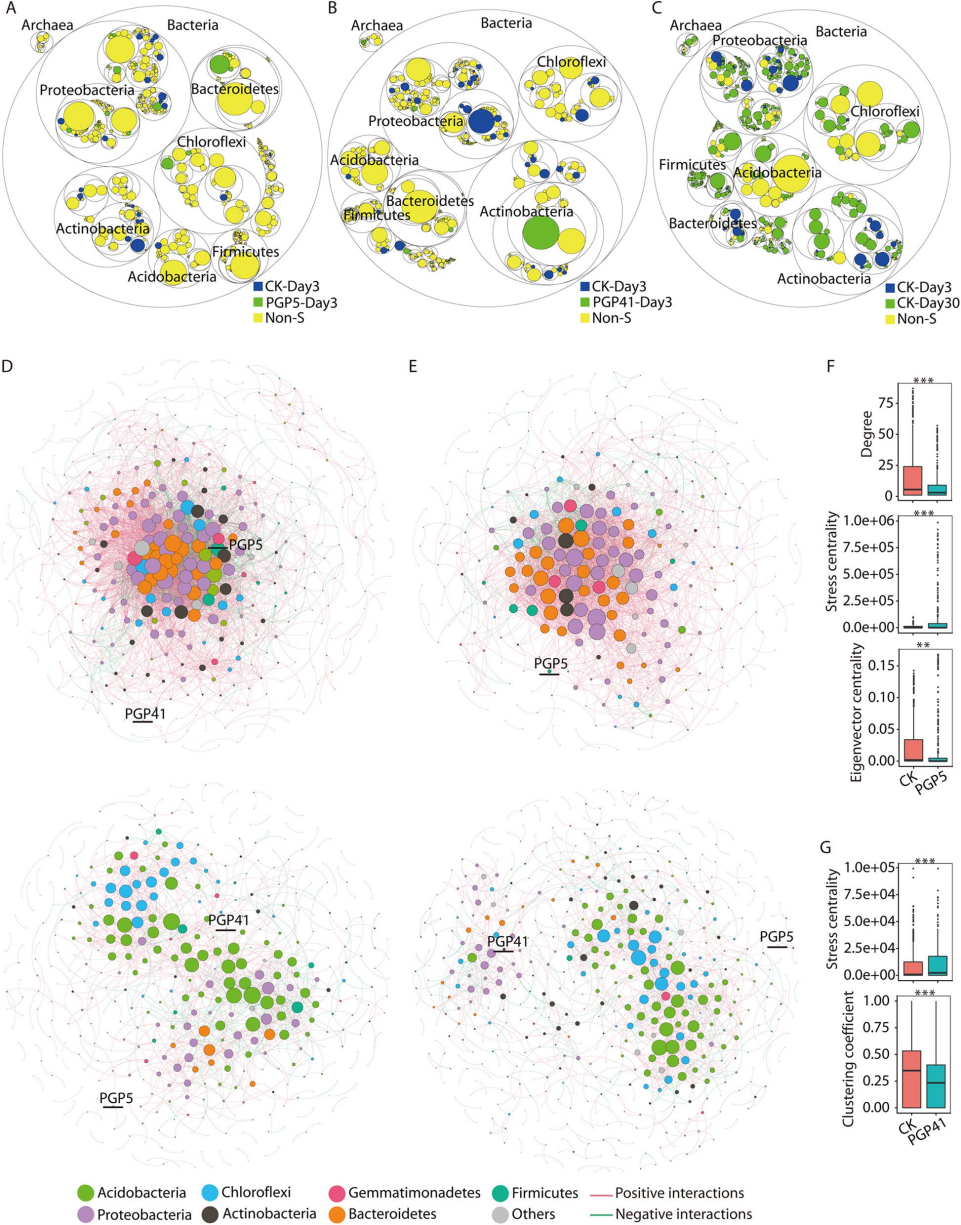
Taxonomic variation in the rhizosphere microbiome induced by inocula. **A**–**C** Taxonomic differences in rhizosphere microbiomes for comparisons of PGP5-Day 3 vs. CK-Day 3 (**A**), PGP41-Day 3 vs. CK-Day 3 (**B**), and CK-Day 30 vs. CK-Day 3 (**C**). The largest circles represent domains (Bacteria and Archaea) and the inner circles refer to phylum, class, family, and genus. The circle sizes represent the relative abundances of taxa at different taxonomic levels. Blue circles represent significantly enriched genera in CK-Day 3; green circles represent significantly enriched genera in PGP5-Day 3, PGP41-Day 3, and CK-Day 30 in panels **A**, **B** and **C** respectively; and yellow circles represent non-significantly enriched (Non-S) genera. **D** Co-occurrence networks of the non-inoculated microbiome in early (top) and late (bottom) phases. **E** Co-occurrence networks of the PGP41-inoculated microbiome in early (top) and late (bottom) phases. Nodes refer to OTUs; edges refer to significant correlations. The color of each node indicates the phylum; the size of each node is proportional to the degree. The OTUs represent strains PGP5 and PGP41 (with 100% identity) are labeled in the networks. **F**, **G** Differences in node-level properties of the co-occurrence networks for comparisons of non-inoculated vs. PGP5-inoculated microbiomes (**F**), and non-inoculated vs. PGP41-inoculated microbiomes (**G**) in the early phase. Significant differences were detected between samples in the early phase (Wilcoxon test, ***P < 0.001; **P < 0.01; *P < 0.05) while no significant differences were detected between samples in the late phase

Although root residence and development were the main factors influencing the rhizosphere microbiome, inoculation-induced changes in the rhizosphere microbiome were detected in the early phase. In total, 58 genera with significantly different abundances were detected in PGP5-Day 3 and PGP41-Day 3 compared to CK-Day 3 (Fig. 2A, B). Hierarchical clustering based on relative abundances of these genera showed three clusters (Fig. S2). For PGP41-specific abundant genera, all day 0 samples clustered together, PGP41-Day 3 formed the second cluster, and the remaining samples grouped in a third cluster (Fig. S2). A similar clustering pattern was found for PGP5-specific genera, except that PGP5-Day 3, 7, and 15 formed the second cluster (Fig. S2). These results suggest that the differentially abundant genera in PGP41-Day 3 or PGP5-Day 3 mainly functioned in the early phase (if at all) and then were restored to CK levels in the late phase. Besides, profound differences were detected between early and late phases in co-occurrence network analyses (Fig. 2D–G and Fig. S3). In general, early phase networks were much more complex, connected, and close than late phase networks, reflected by the higher total links, average degree, and average clustering coefficient, as well as the lower average path lengths and harmonic geodesic distances (Fig. S3). In addition, there were marked differences in the networks between CK and PGP5 or PGP41 in the early phase but not in the late phase, as confirmed by the node-level features (Fig. 2F, G). Furthermore, inoculation with PGP5 or PGP41 remarkably decreased the negative correlations in the early phase, from 19.7% (CK) to 13.6% (PGP5) and 10.5% (PGP41) (Fig. 2D, E and Fig. S3). No marked difference in negative correlations was detected in the late phase, with 26.4%, 28.6%, and 27.2% for CK, PGP5, and PGP41, respectively. The increased negative correlations in the late phase compared to the early phase might result from the recruitment of specific bacteria by roots, which exclude non-related bacteria. Taken together, the results indicate the persistent effects of recruitment by roots on rhizosphere bacteria while inoculations mainly influence the rhizosphere microbiome in the early phase at the taxonomic level.

**Fig. 3:**
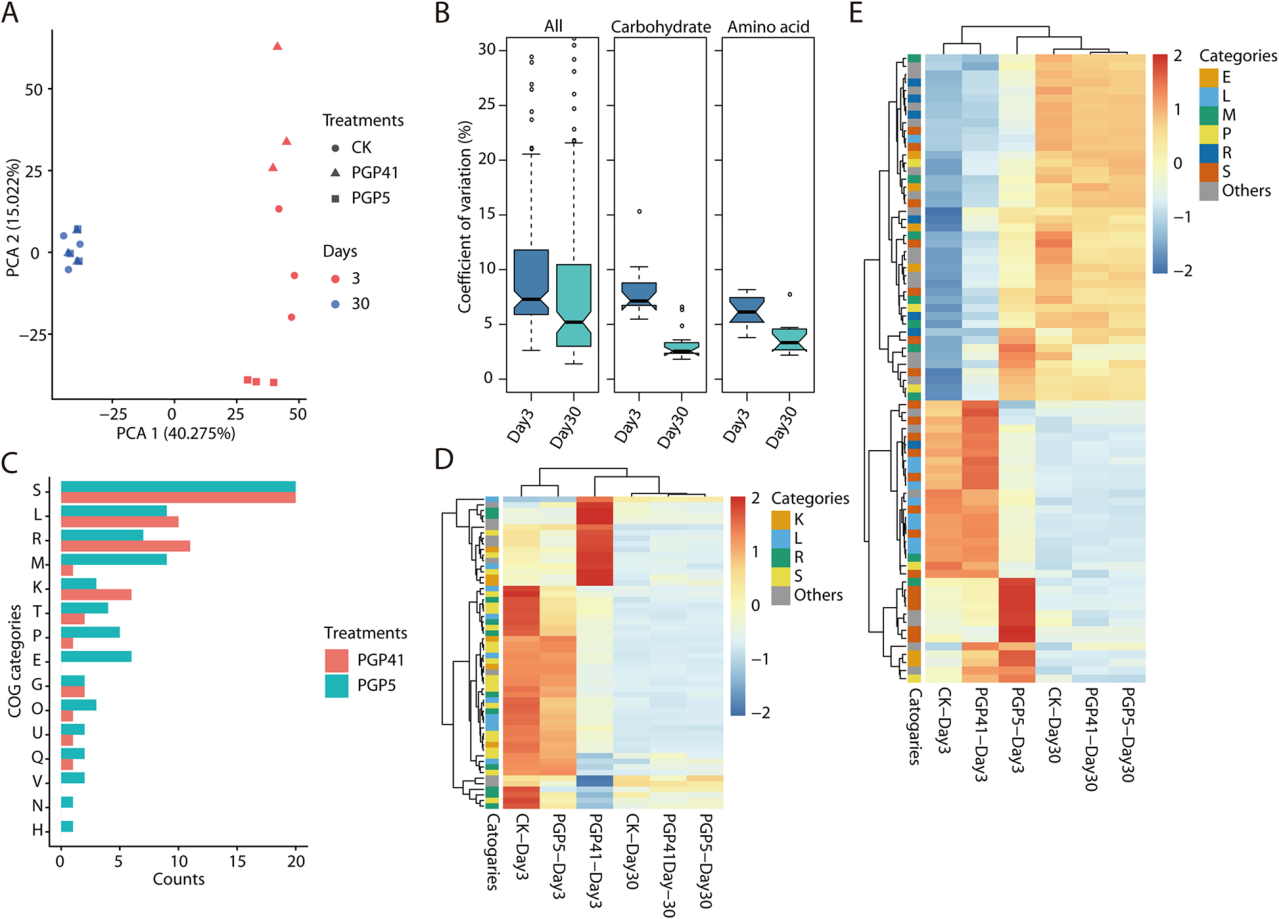
Functional variation in the rhizosphere microbiome. **A** PCA showing variation in the microbiome induced by inoculation in the early phase rather than the late phase. **B** Distributions of coefficients of variation for KEGG categories detected in metagenomes for all samples in the early (day 3) and late (day 30) phases; (left) all KEGG categories; (middle) KEGG categories related to carbohydrate metabolism; (right) KEGG categories related to amino acid metabolism. **C** Abundance of significantly changed COG categories in PGP41- and PGP5-inoculated microbiomes compared to CK at day 3. **D**, **E** Heatmaps depicting the average abundances of significantly changed COG categories in **D** PGP41- and **E** PGP5-inoculated microbiomes compared to CK. COG categories are abbreviated as follows: E, amino acid transport and metabolism; G, carbohydrate transport and metabolism; H, coenzyme transport and metabolism; K, transcription; L, replication, recombination, and repair; M, cell wall/membrane/envelope biogenesis; N, cell motility; O, posttranslational modification, protein turnover, and chaperones; P, inorganic ion transport and metabolism; Q, secondary metabolites biosynthesis, transport, and catabolism; R, general function prediction only; S, function unknown; T, signal transduction mechanisms; U, intracellular trafficking, secretion, and vesicular transport; and V, defense mechanisms

Metagenomic analyses were performed to gain insights into the functional differences between rhizosphere microbiomes. Day 3 and day 30 samples were selected to represent the early and late phases, respectively. In principal component analysis (PCA) of the functional profiles, day 3 samples were separated from the day 30 samples on the first coordinate axis, and PGP5 and PGP41 samples were separated from CK at day 3 on the second coordinate axis (Fig. 3A). However, at day 30, no separation was detected among PGP5 and PGP41 samples and CK (Fig. 3A). All genes annotated with KEGG categories showed higher variation (measured as a coefficient of variation across all samples) in day 3 samples than in day 30 samples (Fig. 3B). Significant variation was observed when only carbohydrate metabolic categories or amino acid metabolic categories were included in the analyses (Fig. 3B). For day 3 samples, totals of 55 and 77 COGs of proteins with significantly different abundances were identified in PGP5–CK and PGP41–CK comparisons, respectively (Fig. 3C–E). However, these COGs showed similar abundances between samples at day 30 (Fig. 3D, E). Furthermore, we detected no COG or KEGG categories with significantly different abundances in PGP5–CK or PGP41–CK comparisons at day 30. Together, these results indicate that functional-level variation in the rhizosphere microbiome induced by inoculation treatments was limited to the early phase.

### Microbiome-induced changes in root gene expression are selectively maintained into the late phase

**Fig. 4:**
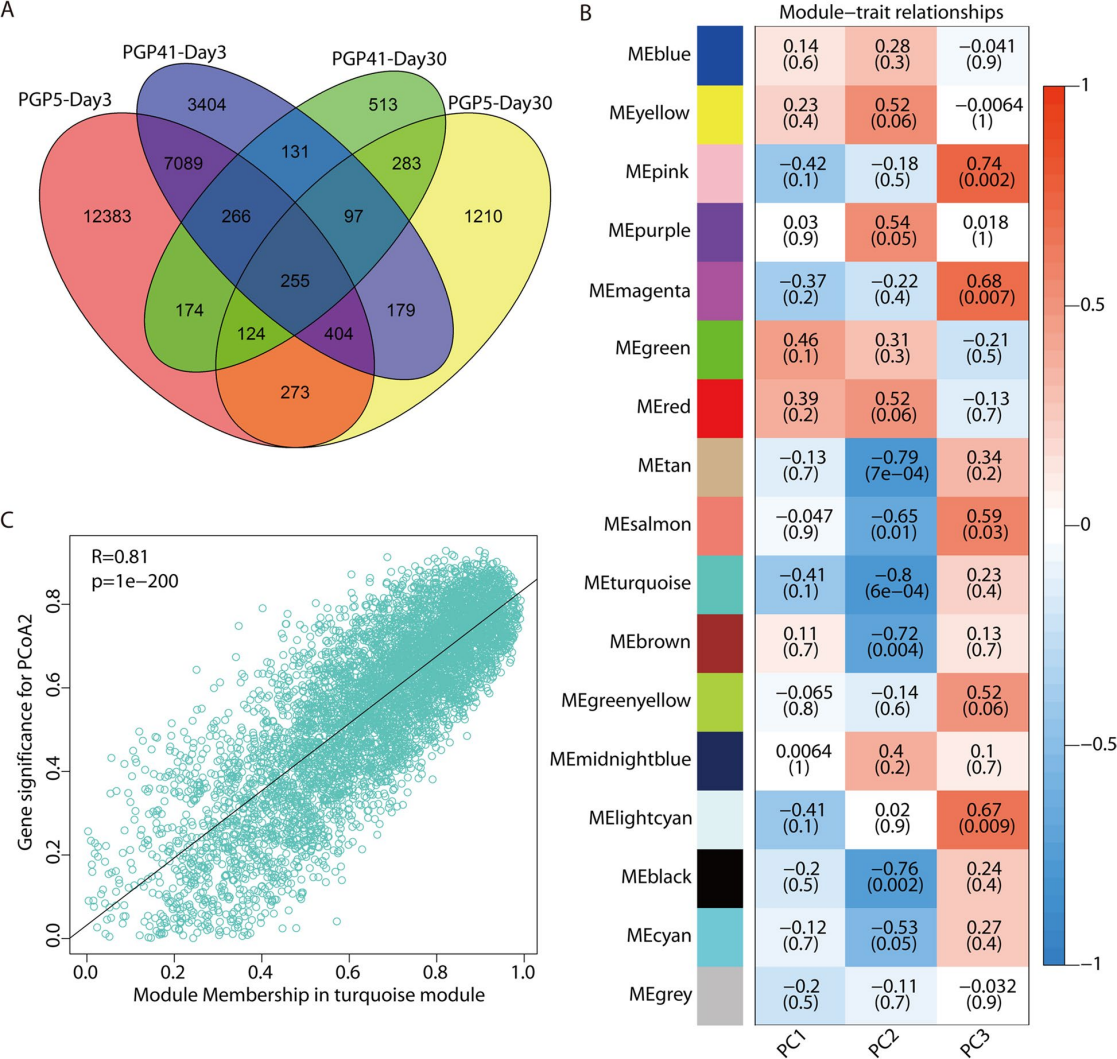
Changes in gene expression profiles in roots induced by the rhizosphere microbiome. **A** Venn diagram of DEGs in inoculated samples in the early and late phases. The DEGs were identified by comparisons of non-inoculated (CK) vs. inoculated (PGP5/PGP41) samples (FDR < 0.05; fold change ≥ 2). **B** WGCNA showing significant correlation between module eigengenes (ME) and traits of the rhizosphere microbiome represented by PCoA1–3 in Fig. 1G. Each module corresponds to a distinct color shown in the left color column; correlation coefficients are shown in the right color bar. The numbers in cells refer to the correlation coefficient and the corresponding *P* value (numbers in brackets) between modules traits. **C** A scatterplot of gene significance (GS) versus module membership (MM) in the most significant module (turquoise module), with a correlation coefficient of 0.81 and *P* < 2e–200

We used RNA sequencing (RNA-seq) to establish differential patterns of gene expression between plants grown in inoculated and non-inoculated soils in both the early and late phases. PCA showed that the two phases have widely different gene expression patterns (Fig. S4), in agreement with the results based on the rhizosphere microbiome. At day 3, totals of 20,968 and 11,825 differentially expressed genes (DEGs) were detected in the PGP5–CK and PGP41–CK comparisons, respectively (Fig. 4A), indicating a thorough change in gene expression elicited by inoculation and/or the altered rhizosphere microbiome in the early phase. Far fewer DEGs were detected at day 30, with 2825 and 1843 in the PGP5–CK and PGP41–CK comparisons, respectively. Interestingly, 37.4% and 40.6% of the DEGs at day 30 were also differently expressed at day 3 for PGP5 and PGP41, respectively (Fig. 4A). Gene Ontology (GO) enrichment analyses of DEGs showed that most of the enriched terms (64% and 76% for PGP5 and PGP41, respectively) in the late phase were also enriched in the early phase (Tables S1 and S2). These results show that the DEGs in the late phase might result from the induction of inoculation in the early phase, as no significant differences were detected between rhizosphere microbiomes at day 30. Besides, GO enrichment analyses revealed that the DEGs were involved in “hydrogen peroxide metabolic process,” “carbohydrate metabolic process,” and “cell wall organization,” which are related to the interactions between plants and microbes [40–43]. The faster and stronger response of roots in the early phase could be caused by priming, which defines the post-challenge primed state, leading to increased resistance [44].

The weighted gene coexpression network analysis (WGCNA) was used to determine modules of DEGs with highly correlated expression patterns and associations of root gene expression with rhizosphere microbiomes. A total of 16 coexpression modules were identified (Fig. 4B). We further investigated the relationships between the module eigengenes and traits of the rhizosphere microbiome represented by the three main principal components in PCoA of the microbial community shown in Fig. 1G. Eight modules (*P* < 0.05) were significantly associated with PCoA2 or PCoA3, and another five modules showed weak associations (*P* < 0.06) with only three modules without correlation with the three main principal components (Fig. 4B). The results indicate that the expression of DEGs in roots was highly related to the rhizosphere microbiome, particularly turquoise modules (Fig. 4C, *R* = 0.81, *P* < 0.001). Combined with results showing most DEGs and their significantly enriched functions in the late phase were also detected in the early phase, these results indicate that microbiome-induced changes in root gene expression were selectively maintained into the late phase.

### Modification of DNA methylation in response to inoculation affects gene expression in roots

**Fig. 5:**
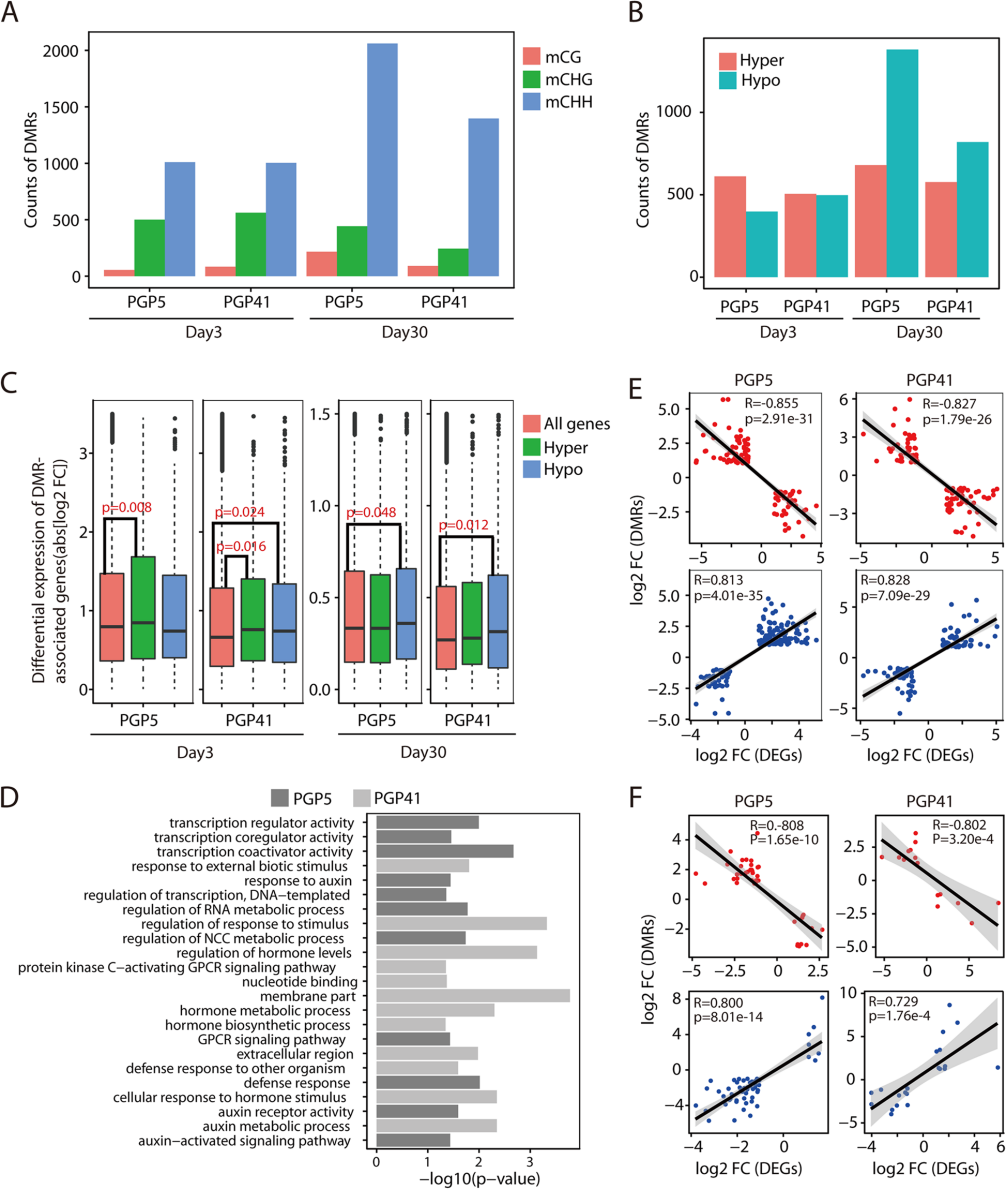
Variation in DNA methylome profiles and correlations with gene expression. **A** Numbers of DMRs detected in different contexts. **B** Numbers of hyper- or hypomethylated DMRs detected in different samples. **C** Differential expression levels of all genes (red) and hyper- (green) or hypomethylated (blue) DMRs. Wilcoxon *P* values are reported. **D** GO enrichment analyses of DMR-associated genes. NCC, nucleobase-containing compound; GPCR, G protein-coupled receptor. **E**, **F** Scatter plots of the changes (log2 fold change) in DNA methylation (*Y*-axis) against the changes in gene transcript abundance (*X*-axis) of overlapping DEGs and DMRs at day 3 (**E**) and day 30 (**F**). The overlapping genes were divided into two sets showing positive and negative relationships respectively. And the negative and positive correlations are displayed in red and blue colors, respectively

The genes involved in maintaining DNA methylation showed significantly different expression patterns between inoculated and non-inoculated roots in early phases but not in the late phase (Fig. S5). To test whether inoculation affected DNA methylation in roots, WGBS profiling of DNA methylation was performed. In total, 14–19 million methylated cytosines (mCs) in each sample were identified. Of these, 7–9 million mCs were in the CG context, 5–6 million were in the CHG context, and 3–4 million were in the CHH context (Fig. S6). Many more differentially methylated regions (DMRs) were identified in the CHH context compared to those in CG (9–20 fold) and CHG (2–6 fold) contexts (Fig. 5A); therefore, we only focused on DMRs in the CHH context. At day 3, we identified 1010 and 1002 DMRs in PGP5–CK and PGP41–CK comparisons, respectively, 163 (16.1%) and 108 (10.8%) of which were also identified at day 30 (Fig. S6). These results indicate that changes in DNA methylation in certain regions were maintained from the onset to the late phase during the plant–microbe interactions. In PGP5-Day 3, hypermethylated DMRs were dominant, while almost equal amounts of hypo- and hypermethylated DMRs were found in PGP41-Day 3 (Fig. 5B). However, at day 30, hypomethylated DMRs became dominant in both PGP5 and PGP41 samples (Fig. 5B). Correspondingly, hypermethylated (dominant type) DMRs in PGP5-Day 3 showed higher levels of differential transcript abundance compared to all genes (*P* < 0.01; Fig. 5C), whereas hypomethylated DMRs exhibited similar levels of differential transcript abundance relative to all genes (Fig. 5C). Significant differences were also detected for the dominant type of DMRs in PGP5-Day 30 (*P* < 0.05) and PGP41-Day 30 (*P* < 0.05), while the non-dominant type showed similar levels (Fig. 5C). For PGP41-Day 3, where no dominant DMR type was detected, both hypo- and hypermethylated DMRs showed differential transcript abundance (*P* < 0.05, Fig. 5C). The relationship between the dominant DMR type and transcript abundance suggests that DNA methylation modification induced by inoculation might be involved in the regulation of gene expression.

We further tested the influence of DMRs on gene expression. First, we analyzed the correlation between altered gene expression and DNA methylation levels based on overlapping DEGs and DMRs. At both day 3 and day 30, strong positive or negative relationships were detected between the fold changes in gene expression and DNA methylation (*R* > 0.7 and *P* < 0.001, Fig. 5E, F). Furthermore, GO analyses of DMRs in the late phase revealed enrichment of genes involved in the regulation of transcription, regulation of hormone levels, defense response, nucleotide binding, and the G protein-coupled receptor signaling pathway (Fig. 5D). All of these functions are related to gene expression regulation. In addition, among the DEGs in the turquoise module that was most significantly related to inoculation (Fig. 4C), 420 (7.6%) genes were identified as DMRs. Together, these data indicate that gene transcription was at least partially controlled by DNA methylation in the interaction between strains PGP5/PGP41 and *P*. *americana*.

### Changes in DNA methylation are involved in inoculation-induced growth promotion of P. americana

The abundance of inoculated strains in rhizosphere soils and roots was calculated by quantifying the copy numbers of their 16S rRNA gene by quantitative PCR (qPCR) (Fig. S7). The inoculation significantly increased abundances of strain PGP5 and PGP41 in rhizosphere soils at early state (day 3 and 7), and the abundances of both inocula rapidly decreased to the same level in control soils. No significant difference was detected in the inoculated roots compared to controls (the non-inoculated roots) for either strain PGP5 or PGP41. The results indicated both inocula were present in the rhizosphere soils at early stage which were eliminated from rhizosphere soils at late stage and no colonization of inocula in roots. The conclusion was further verified by fluorescence in situ hybridization (FISH), green fluorescent protein (GFP)-tagged strain, and 16S rRNA gene amplification. The FISH signals of both inocula in rhizosphere soils were detected at day 3 but were absent at day 30 (Fig. S8 and S9), showing the elimination of inocula from rhizosphere soils. Similar results were got by the GFP-tagged strain (Fig. S10). Neither FISH signal (Fig. S11) nor GFP-tagged strain (Fig. S10) was detected in inoculated roots. Besides, the amplification was successful with PGP5- and PGP41-specific 16S rRNA gene primers from DNA of strain PGP5 and PGP41 respectively, but it was failed for root DNA (Fig. S11). These results suggested no colonization of inoculated strains in roots of *P. americana*. To test whether variation in the rhizosphere microbiome is necessary for plant growth promotion by PGPB, we used sterilized soils for plant development and inoculation treatments. No microbe was isolated from the sterilized soils (Fig. S13), showing the soils were sterilized completely. Inoculation of each strain significantly promoted plant growth in sterilized soils (Fig. S14 and S15). Together, these results indicate that neither changes in the rhizosphere microbiome nor colonization of inocula in roots is necessary for plant growth promotion.

**Fig. 6:**
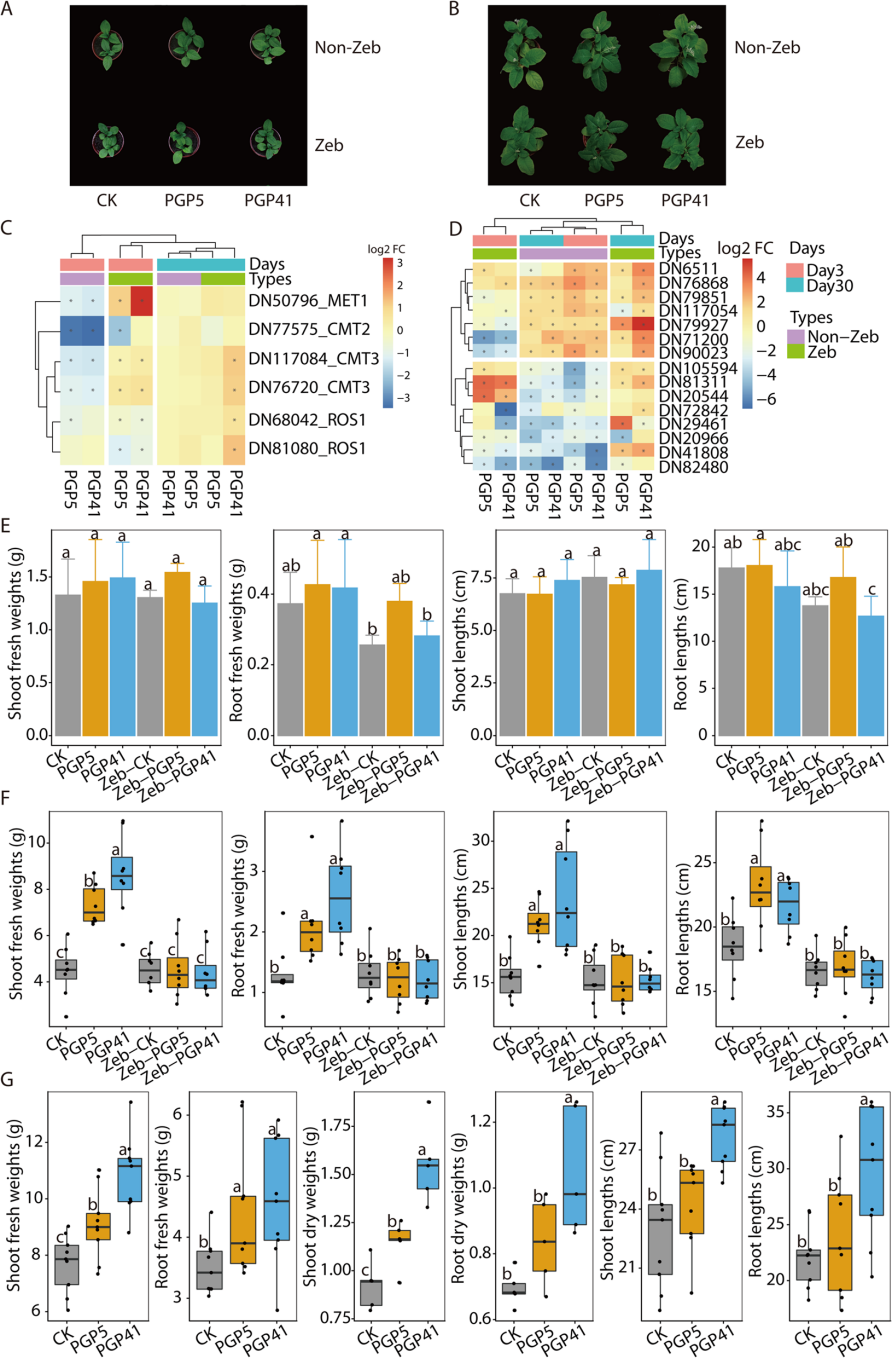
Treatments with DNA methylation inhibitor disrupt *P*. *americana* growth promotion induced by inoculation with strains PGP41 and PGP5. **A**, **B** Aerial part of *P*. *americana* under different treatments at day 3 (**A**) and day 30 (**B**). The plants were treated with zebularine (Zeb), a DNA methylation inhibitor, to investigate the role of DNA methylation in the growth-promoting process. Zeb, Zeb-treated samples; Non-Zeb, samples without Zeb treatment. **C**, **D** Heatmaps based on relative transcript abundances (log2 fold change detected by comparisons of treated vs. control samples) of genes involved in maintaining DNA methylation (**C**) and genes randomly selected from the overlapping DEGs and DMRs showed in Fig. 5 (**D**). For both the DNA methylation-related genes and the DEG-DMRs overlapped genes, Zeb treatments caused misregulation of these gene expressions in Zeb-treated roots compared to non-Zeb-treated plants (some upregulated genes in non-Zeb-treated plants showed downregulation in Zeb-treated plants and vice versa). Red, increased transcript abundance; blue, decreased transcript abundance. The relative transcript abundances of Zeb samples were obtained by qRT-PCR; the primers used are listed in Table S3. Asterisks indicate significant differences (*P* < 0.05). **E**, **F** Comparison of inoculation-induced *P*. *americana* growth promotion with and without Zeb treatment at day 3 (**E**, *n* = 3) and day 30 (**F**, *n* ≥ 8). The weights and lengths of both shoots and roots are shown. Different letters indicate significant differences (Duncan’s test, *P* < 0.05). **G** The inoculation-induced *P. Americana* growth promotion by strains PGP41 and PGP5 were detected at day 60 (*n* ≥ 8), showing long-time growth promotion induced by inoculations at early phase

To investigate the role of DNA methylation in inoculation-induced growth promotion of *P*. *americana*, we treated seedlings of *P*. *americana* with Zeb which is a DNA methylation inhibitor (Fig. 6A, B). After treatment, we inoculated both Zeb-treated and non-Zeb-treated *P*. *americana*, and the expressions of genes related to the inoculation-induced growth promotion were detected (Fig. 6C, D). Without Zeb treatment, genes involved in maintaining DNA methylation showing significantly different expression patterns between inoculated and non-inoculated roots in the early phase but not the late phase (Fig. 6C). However, with Zeb treatment, these genes showed misregulation of gene expression in Zeb-treated roots compared to non-Zeb-treated plants (Fig. 6C), showing that the Zeb treatment disrupted inoculation-induced DNA methylation patterns in the Zeb-treated plants. To further verify the effect of disruptions of Zeb-induced DNA methylation on expressions of genes related to inoculation-induced growth promotion, we analyzed expressions of 15 genes randomly selected from the overlapping DEGs and DMRs (Fig. 6D). Not surprisingly, misregulation of gene expression in Zeb-treated roots compared to non-Zeb-treated plants was detected among these genes. Together, the results suggested that Zeb treatments could disrupt the inoculation-induced gene expression patterns by altering the DNA methylation patterns. Consistently, with inoculation treatments, non-Zeb-treated *P*. *americana* (PGP5 and PGP41) showed significantly increased biomass of both roots and shoots compared to CK samples at day 30 (Fig. 6F), but not at day 3 (Fig. 6E), suggesting that inoculation induced plant growth promotion in the absence of Zeb treatment. More effects of inoculation on the growth of *P*. *americana* studied by both pot and filed experiments are described in Supplementary Materials (Fig. S16 and S17). However, at day 30, no significant difference in biomass was detected between Zeb + inoculation treatments (Zeb-PGP5 and Zeb-PGP41) and Zeb-only treatments (Zeb-CK) (Fig. 6F). Furthermore, the Zeb-treated control samples (Zeb-CK) and untreated CK samples showed no differences in biomass at day 30. These results indicate that the inoculum-induced plant growth promotion was greatly weakened by Zeb treatment. Besides, similar results for Zeb-treated and PGPB-inoculated plants in sterilized soils, i.e., both inoculants promoted plant growth while the Zeb treatment resulted in both inoculants losing the ability to induce plant growth promotion (Fig. S15). Together, these results suggested that the inoculation-induced growth promotion of *P*. *americana* is at least partially mediated by change in DNA methylation. Furthermore, we also detected the growth promotions of *P. americana* induced by inoculations at 60 days after inoculation (Fig. 6G), which suggested the altered DNA methylation elicited by the inoculum in the early phase has a long-term effect for inoculation-induced plant growth promotion after the inoculum was eliminated in soils.

## Discussion

Microbial communities may be functional drivers of their host plants, with roles including expanding plant metabolic capabilities, facilitating nutrient acquisition, and providing biotic/abiotic stress tolerance. In the past decade, to solve agriculture-associated problems, strategies based on plant microbiome engineering have been proposed [5–8]. However, taking plant-beneficial microorganisms from discovery to agricultural application remains challenging, as the mechanisms underlying the interactions between beneficial strains and plants in native soils are still largely unknown. As many studies have shown that strains introduced to manipulate microbiomes are usually eliminated in soils and do not persist at functionally meaningful abundances [8], many efforts have focused on improving the persistence of inocula, such as using genetically modified strains or functional consortia instead of a single functional strain [5, 45, 46]. In this study, we found that DNA methylation mediated the inoculation-induced plant growth promotion process and remained functional after elimination of the inoculum from the rhizosphere microbiome. These results not only provide important insights into the mechanisms underlying microbe–plant interactions but also offer strategies for plant microbiome engineering beyond the perspective of maintaining inoculum persistence in soils.

It is well documented that plants affect rhizosphere microbiomes [8, 9, 47]. The explosive development of high-throughput sequencing technologies and analysis tools greatly improves our ability to explore the influence of PGPB inoculation and plant development on the other microorganisms [48, 49]. We analyzed rhizosphere microbiome dynamics during the development of *P*. *americana* after inoculation and compared the influences of plant recruitment and PGPB inoculation. Our results indicate that root residence and recruitment are the main factors driving variation in the rhizosphere microbiome. Meanwhile, the inoculation-induced influences on the bacterial community were limited to the early phase. The reduced influence of inoculation on the rhizosphere microbiome might first result from the elimination of the inoculum in soils, after which the early influences were attenuated or eliminated in the late phase by recruitment from plant roots. In addition, both inocula promoted growth of *P*. *americana* in sterilized soils without colonizing in roots. These results indicate that neither variation in the rhizosphere microbiome nor root colonization by inocula is the main factor mediating growth promotion of *P*. *americana*.

Although several studies have demonstrated that DNA methylation plays a key role in the regulation of gene expression in plants responding to phytopathogenic bacteria [20, 23], few studies have focused on DNA methylation dynamics during the response to PGPB stimuli. In plant–phytopathogen interactions, hypomethylation has been reported in interactions between plants and phytopathogens [20, 23, 50]. In our study, hypermethylation in the early phase and hypomethylation in the late phase were predominant in plants inoculated with PGP5. Meanwhile, equal amounts of hypo- and hypermethylated DMRs were detected in the early phase in the plant–PGP41 interaction, with hypomethylation being predominant in the late phase. The conversion of dominant DMR types between the early and late phases revealed the dynamics of DNA methylation levels during plant–PGPB interactions. These results suggest the possibility that plants can distinguish between different types of microbes to establish different interactions based on epigenetic variation.

Methylated cytosines occur in almost the entirety of plant genomes, including gene bodies, sequences flanking gene bodies (such as upstream promoter regions and downstream untranslated regions), and transposable elements [22, 29, 51]. Although the roles of DNA methylation within gene boundaries or transposable elements in regulation of gene expression have been frequently documented, the functional roles of DNA methylation in gene bodies are still unknown [51]. In *Arabidopsis*, one-third of methylated genes occur in transcribed regions, suggesting that methylated genes may account for a large proportion of gene-coding regions in plants [52]. In addition, by mapping DNA methylation in the *Arabidopsis* genome, an interwoven relationship between gene transcription and DNA methylation has been proposed: transcription strongly influences gene methylation while gene methylation in turn affects transcription [18]. Furthermore, a strong correlation between variation in transcript abundance and modification of DNA methylation in gene bodies rather than in non-genic regions has been detected [20]. These results indicate possible functions of gene body methylation in gene expression regulation. Here, we used a non-model plant to study the epigenetic regulation of gene expression during plant–PGPB interactions in natural soils. Without an available genome, we focused only on genic methylation. Our results showed that gene expression could be influenced by DNA methylation modifications in gene bodies, and plant–PGPB interactions are involved in these modifications. In future studies, comprehensive analyses of DNA methylation in the entire genome are needed to systematically document the functional roles of DNA methylation in different regions.

**Fig. 7:**
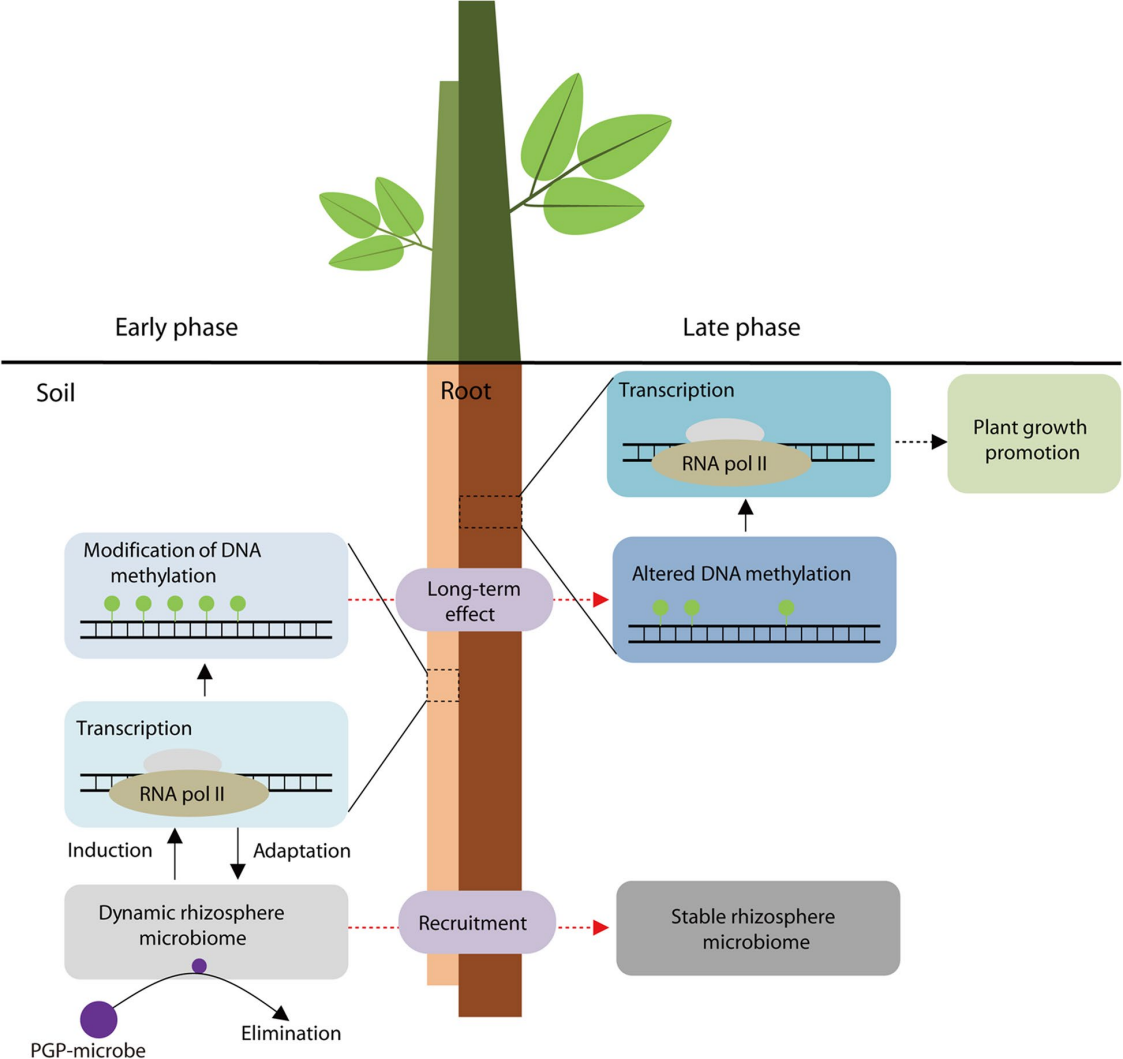
Schematic representation of the two-step interaction between PGPB and plants mediated by DNA methylation and root recruitment. Purple circles indicate the PGPB inoculum. Green circles represent methylated cytosines. In the early phase, inoculation with PGPB induced variation in the rhizosphere microbiome. Plants adapt to the dynamic rhizosphere microbiome through comprehensive changes in transcription profiles, including DNA methylation-related genes, which results in the modification of DNA methylation. The influences of inocula on the rhizosphere microbiome weaken along with the elimination of the inoculum from the rhizosphere microbiome. In the late phase, the altered DNA methylation regulates gene expression to facilitate plant growth, and a stable rhizosphere microbiome is assembled by recruitments of roots

Two clearly separated phases were detected during the interaction between the rhizosphere microbiome and plants. In the early phase, the rhizosphere microbiome was dramatically influenced by root residence and recruitment, while only moderate changes were detected in the late phase, suggesting that a stable root–microbiome system was formed. This result is consistent with the two-step selection process by which plants recruit rhizosphere microbes from soil: (1) root exudates improve the growth of some soil bacteria, leading to a shift of the microbial community, and (2) plant selection fine-tunes the bacterial communities thriving on the rhizoplane [9]. However, the dynamics of plant responses to variation in the rhizosphere microbiome are lacking in the current two-step process. In this study, we complemented the process with plant response dynamics based on transcriptome and methylome analyses, which also suggested two distinct stages of root responses to the rhizosphere microbiome. Together, a model of a two-step process of interactions between the microbiome and plant mediated by DNA methylation and root recruitment was proposed (Fig. 7). Specifically, the early influence of the inoculum on the rhizosphere microbiome was attenuated in the late phase by root recruitments after elimination of the inoculum. Meanwhile, altered DNA methylation elicited by the inoculum in the early phase might regulate gene expression after the inoculum was eliminated in the late phase, showing a long-term effect. The model comprehensively documents the dynamic regulation processes of both the rhizosphere microbiome and plant.

## Conclusions

Our study provides intriguing insights into microbe–plant interactions and highlights the importance of DNA methylation modifications in roots in response to PGPB, presenting a new mechanism that PGPB-induced DNA methylation modification in roots promotes the plant growth. In addition, these epigenetic modifications remained functional even after elimination of the inoculum from the microbiome. These results offer new strategies for microbiome manipulation to promote plant growth through the application of PGPB. In future studies, more efforts should be taken in uncovering the detailed process of PGPB-induced modifications of DNA methylation at early stage to document whether and which specialized molecules/metabolites produced by PGPB can modulate root DNA methylation, which would be very useful for the commercialization of PGPB in field applications.

## Materials and methods

### Plant, PGPB, and soil materials

The soil used in this study was surface soil (0–15 cm depth) collected from Nanjing (32° 09′ N, 118° 57′ E), China. To remove plant debris and stones, the soil samples were passed through a 4-mm sieve. The samples were transported to the laboratory and stored at 4°C until further use.

Two PGPB strains, *Bacillus* sp. PGP5 and *Arthrobacter* sp. PGP41, were used in this study. The strains were separately incubated in Luria-Bertani broth (LB) at 150 rpm and 30°C for 24 h. The cells were harvested, washed three times with sterile water, and resuspended in sterile 0.9% NaCl solution. The cell suspension was adjusted to a final optical density at 600 nm of 1.0 (corresponding to approximately 0.29 × 10^9^ and 1.47 × 10^9^ CFU/mL for strains PGP5 and PGP41, respectively).

To eliminate effects associated with genetic variations of plants on the plant–microbiome interactions, isogenic pokeweeds were used in this study. Approximately 200 seeds of *P. americana* were firstly harvested from one single plant near a lead and zinc ore smeltery in Jishou, Hunan Province, China (28° 17′ N, 109° 45′ E [37]) to eliminate effects associated with genetic variations. Then, these seeds were planted in pots and placed in a greenhouse with a 13/11 h day/night photoperiod at 25°C to eliminate maternal effects. The harvested seeds from greenhouse were used in our following experiments. The obtained *P*. *americana* seeds were soaked in concentrated sulfuric acid for 10 min, followed by a thorough water rinse, and then sown in vermiculite. After germination, the seedlings were transferred to a vessel containing 1.2 L of Hoagland nutrient solution. Seedlings of *P*. *americana* at the four-leaf stage were inoculated with the two PGPB strains. The inoculation was performed in separate pots (upper caliber, 14 cm; lower caliber, 10.5 cm; depth, 11.5 cm) with 1 kg soil in each pot. The roots were first dipped in the inoculum (OD_600_ = 1.0) or sterile water (control) for 5 min and then immediately transferred to pots. A 50-mL suspension of the inoculum was added to soil samples in each pot. Pots were randomly placed in a greenhouse with a 13/11 h day/night photoperiod at 25°C.

### Experiment design

The experiment included two inoculated treatments and one non-inoculated control treatment (Fig. S1). The treatments were as follows: PGP5 (inoculation with strain PGP5), PGP41 (inoculation with strain PGP41), and CK (same volume of sterile water added to soil instead of inoculum). All treatments were performed in triplicate. At 0, 3, 7, 15, 21, and 30 days after inoculation, the plants and rhizosphere soils were sampled. Roots were collected and gently shaken to remove the loosely adhered soil, after which the rhizosphere soil samples were collected by removing the remnant soil with a fine sterile brush. Samples were frozen in liquid nitrogen and stored at –80°C until further analyses.

Sterilized soil was used to investigate the role of the rhizosphere microbiome in the growth-promoting process (Fig. S1). All the treatments were the same as mentioned above except for soils which were sterilized (121°C, 2 h). Besides, seedlings of *P*. *americana* were treated with Zeb to investigate the role of DNA methylation in the process inoculation-induced growth promotion (Fig. S1). *P*. *americana* seedlings were grown in Hoagland nutrient solution containing Zeb (3 μM) for 7 days before transfer to pots for further inoculation treatments. Control plants were treated with distilled water and cultivated under the same conditions. The inoculation treatments were the same as mentioned above. To monitor the presence of the inoculant strains in rhizosphere soils and roots, the combination of qPCR, FISH, GFP-tagged strain, and 16S rRNA gene amplification were used. The detailed methods are described in Supplementary Materials.

### 16S rRNA amplicon sequencing

In total, 54 sequencing libraries were constructed, and the details of sequencing library construction are described in Supplementary Materials. The Illumina raw reads were quality-filtered following previously described criteria [45]. Chimeric sequences were identified and removed using UCHIME [53]. The OTUs were analyzed using the UPARSE pipeline [54] and sequences were assigned to OTUs at a 3% dissimilarity cutoff. The Ribosomal Database Project classifier [55] was used for taxonomic annotation of each OTU at the 70% threshold. The α- and β-diversity were analyzed using the QIIME pipeline [56].

### Metagenomic analyses

Metagenomic analyses were used to infer differences in the functional potential of the rhizosphere microbiome with and without inoculation. Soil samples were collected in the early (day 3) and late (day 30) phases for metagenomic analyses. Metagenomic shotgun sequencing was performed at Biozeron Biotechnology Co. on the Illumina HiSeq 2500 platform with 150 bp paired-end technology. Raw reads were filter-trimmed using Trimmomatic [57] to remove adapter sequences and low-quality reads, and the resulting clean reads were used for further analyses. Functional annotation was performed by comparing clean reads to the clusters of orthologous groups (COG) and Kyoto Encyclopedia of Genes and Genomes (KEGG) databases.

### RNA sequencing and data analyses

Roots were sampled at 3 and 30 days after inoculation for RNA-seq analyses. The details of sequencing library construction are described in Supplementary Materials. After removing adapters and low-quality reads with Trimmomatic [57], the remaining reads were assembled de novo using Trinity [58] with default parameters. Annotation of unigenes was performed by BLASTx searches against the NCBI non-redundant, Swiss-Prot, KEGG, and COG protein databases. Gene expression was obtained using RSEM [59] and further identification of DEGs was performed on the basis of fragments per kilobase of transcript per million mapped reads (FPKM) values using Cuffdiff [60] with a false discovery rate (FDR) < 0.05 and an absolute of fold change ≥ 2.

### Bisulfite DNA sequencing and data analyses

The details of sequencing library construction are described in Supplementary Materials. The clean reads were mapped to the reference unigenes obtained by RNA-seq using BSMAP [61]. The uniquely mapped reads were reserved to determine the cytosine methylation using a previously described method [62]. For DMR calling, only cytosines covered by at least four reads in a library were used. DMRs were searched using 200-bp bins with a 50-bp step size. Then, Fisher’s exact test was carried out and the *P* values were adjusted using the Benjamini–Hochberg method. Bins with an FDR < 0.05 and a fold change > 1.5 in the methylation level were retained for further analyses. Within selected bins, each cytosine was subjected to Fisher’s exact test and defined as a differentially methylated cytosine (DMC) if the following criteria were met: *P* < 0.01 and fold change ≥ 2 with absolute methylation differences of 0.4, 0.2, and 0.1 for CG, CHG, and CHH contexts, respectively. The bins containing at least seven DMCs were retained. Finally, neighboring DMRs separated by no more than 100 bp were joined together into a larger DMR.

### Quantitative reverse transcription PCR (qRT-PCR)

RNA was reverse-transcribed using the PrimeScript RT reagent kit with gDNA Eraser (Takara Bio). The amplifications were carried out using TB Green Premix Ex Taq (Tli RNaseH Plus) kit (Takara Bio). qRT-PCR was performed on an ABI StepOnePlus real-time PCR system. Actin and beta-tubulin were used as endogenous controls. The relative gene expression levels were calculated using the 2^–∆∆Ct^ method [63]. The sequences of the primers used for qRT-PCR are listed in Table S3.

### Statistical analyses

The PCoA was used to ordinate the microbial community structure by principal coordinates using a Bray–Curtis dissimilarity matrix calculated from the taxonomic abundance matrices. The PCA was used to visualize the community structure using the functional abundance matrices. The PCoA and PCA plots were generated using the vegan package [64] in R. The linear discriminate effect size (LEfSe) program [65] was used to explore the most discriminating OTUs among treatments. Alpha = 0.05 was used in the Wilcoxon rank sum test, and the log value for linear discriminant analyses was set to < 2.0. Differentially abundant COG and KEGG functional entries were determined with the DESeq2 package [66] with criteria of *P* < 0.05 and fold change ≥ 1.5. A random forests approach was used to identify marker OTUs discriminating plant developmental times using the randomForest package [67] in R. One thousand iterations were used to determine the list of OTUs ranked in order of feature importance. The optimal number of marker OTUs was identified using 10-fold cross-validation by the “rfcv” function with five repeats. Networks were constructed for each treatment based on OTU relative abundances in both early and late phases, yielding a total of six networks. The OTUs with more than five sequences per sample were retained for analyses. Network analyses were performed using the Molecular Ecological Network Analyses pipeline [68]. Coexpression networks of 16,316 DEGs with FPKM values > 1 were performed using the weighted gene coexpression network analysis (WGCNA) package [69] in R. The R scripts are publicly available at https://github.com/XXH2021/Epigenetic-memory-in-plant-microbe-interactions.

## Supplementary Information

**Additional file 1: Fig. S1.** Schematic representation of the experimental design. **Fig. S2.** Differential abundances of bacterial communities in inoculated and non-inoculated soils at Day 3 and Day 30. **Fig. S3.** Co-occurrence networks of PGP5 over time, as affected by inoculation. **Fig. S4.** Variation in transcript profiles between the early and late phase. **Fig. S5.** Heatmap based on relative transcript abundances of genes involved in maintaining DNA methylation. **Fig. S6.** Overview of DNA methylation levels and differences in DNA methylation among samples. **Fig. S7.** Comparisons of abundances of strain PGP5 and PGP41 between inoculated and non-inoculated rhizosphere soils and roots by qPCR. **Fig. S8.** Detection of strain PGP41 in rhizosphere soils by FISH. **Fig. S9.** Detection of strain PGP5 in rhizosphere soils by FISH. **Fig. S10.** Detection of strain PGP41 in rhizosphere soils and roots with a GFP-tagged strain. **Fig. S11.** Detection of strains PGP41 and PGP5 in roots by FISH. **Fig. S12.** Detection of strains PGP41 and PGP5 in roots by 16S rRNA gene amplification. **Fig. S13.** Analysis of the effectiveness of soil sterilization. **Fig. S14.** Comparison of inoculum-induced growth promotion of *P*. *americana* in sterilized soils vs. unsterilized soils. **Fig. S15.** Inoculation induced *P. americana* growth promotion in sterilized soils were disrupted by DNA methylation inhibitor. **Fig. S16.** Effects of inoculation of strain PGP5 or PGP6 on contents of Fe, K, P and Mg in *P. americana*. **Fig. S17.** Effects of inoculation of strain PGP5 or PGP41 on height, fresh weight of stems and leaves, number of inflorescences, and fresh weight of inflorescences of *P. americana*. **Fig. S18.** Neighbor-joining tree based on 16S rRNA gene sequences showing the position of strain PGP41 within the genus Bacillus. **Fig. S19.** Variation in the rhizosphere microbiome between Day 3 and Day 30 at the functional level. **Table S1.** GO enrichment analyses of PGP5 DEGs. **Table S2.** GO enrichment analyses of PGP41 DEGs. **Table S3.** Primers used in this study.

## Data Availability

The raw sequencing reads are publicly available under NCBI BioProject accession no. PRJNA607406.

## References

[1] Gallo RL, Hooper LV (2012). Epithelial antimicrobial defence of the skin and intestine. Nat Rev Immunol.

[2] Berendsen RL, Pieterse CM, Bakker PA (2012). The rhizosphere microbiome and plant health. Trends Plant Sci.

[3] Haney CH, Samuel BS, Bush J, Ausubel FM (2015). Associations with rhizosphere bacteria can confer an adaptive advantage to plants. Nat Plants.

[4] Wei Z, Gu Y, Friman V-P, Kowalchuk GA, Xu Y, Shen Q (2019). Initial soil microbiome composition and functioning predetermine future plant health. Sci Adv.

[5] Parnell JJ, Berka R, Young HA, Sturino JM, Kang Y, Barnhart DM (2016). From the lab to the farm: an industrial perspective of plant beneficial microorganisms. Front Plant Sci.

[6] Finkel OM, Castrillo G, Herrera PS, Salas GI, Dangl JL (2017). Understanding and exploiting plant beneficial microbes. Curr Opin Plant Biol.

[7] Orozco-Mosqueda MDC, Rocha-Granados MDC, Glick BR, Santoyo G (2018). Microbiome engineering to improve biocontrol and plant growth-promoting mechanisms. Microbiol Res.

[8] Cordovez V, Dini-Andreote F, Carrion VJ, Raaijmakers JM (2019). Ecology and evolution of plant microbiomes. Annu Rev Microbiol.

[9] Bulgarelli D, Schlaeppi K, Spaepen S, van Themaat EVL, Schulze-Lefert P (2013). Structure and functions of the bacterial microbiota of plants. Annu Rev Plant Biol.

[10] Bashan Y, Puente ME, Rodriguez-Mendoza MN, Toledo G, Holguin G, Ferrera-Cerrato R (1995). Survival of Azospirillum brasilense in the bulk soil and rhizosphere of 23 soil types. Appl Environ Microbiol.

[11] Mallon CA, Poly F, Le Roux X, Marring I, van Elsas JD, Salles JF (2015). Resource pulses can alleviate the biodiversity-invasion relationship in soil microbial communities. Ecology..

[12] Thomas P, Sekhar AC (2016). Effects due to rhizospheric soil application of an antagonistic bacterial endophyte on native bacterial community and its survival in soil: a case study with Pseudomonas aeruginosa from banana. Front Microbiol.

[13] Gadhave KR, Devlin PF, Ebertz A, Ross A, Gange AC (2018). Soil inoculation with Bacillus spp. modifies root endophytic bacterial diversity, evenness, and community composition in a context-specific manner. Microb Ecol.

[14] Mallon CA, Le Roux X, van Doorn GS, Dini-Andreote F, Poly F, Salles JF (2018). The impact of failure: unsuccessful bacterial invasions steer the soil microbial community away from the invader’s niche. ISME J.

[15] Krober M, Wibberg D, Grosch R, Eikmeyer F, Verwaaijen B, Chowdhury SP (2014). Effect of the strain Bacillus amyloliquefaciens FZB42 on the microbial community in the rhizosphere of lettuce under field conditions analyzed by whole metagenome sequencing. Front Microbiol.

[16] Xu XH, Liu XM, Zhang L, Mu Y, Zhu XY, Fang JY (2018). Bioaugmentation of chlorothalonil-contaminated soil with hydrolytically or reductively dehalogenating strain and its effect on soil microbial community. J Hazard Mater.

[17] Wang J, Li Q, Xu S, Zhao W, Lei Y, Song C (2018). Traits-based integration of multi-species inoculants facilitates shifts of indigenous soil bacterial community. Front Microbiol.

[18] Chinnusamy V, Zhu JK (2009). Epigenetic regulation of stress responses in plants. Curr Opin Plant Biol.

[19] Zilberman D, Gehring M, Tran RK, Ballinger T, Henikoff S (2007). Genome-wide analysis of Arabidopsis thaliana DNA methylation uncovers an interdependence between methylation and transcription. Nat Genet.

[20] Dowen RH, Pelizzola M, Schmitz RJ, Lister R, Dowen JM, Nery JR (2012). Widespread dynamic DNA methylation in response to biotic stress. Proc Natl Acad Sci U S A.

[21] Stroud H, Greenberg MV, Feng S, Bernatavichute YV, Jacobsen SE (2013). Comprehensive analysis of silencing mutants reveals complex regulation of the Arabidopsis methylome. Cell..

[22] Secco D, Wang C, Shou H, Schultz MD, Chiarenza S, Nussaume L (2015). Stress induced gene expression drives transient DNA methylation changes at adjacent repetitive elements. Elife..

[23] Hewezi T, Pantalone V, Bennett M, Neal SCJ, Burch-Smith TM (2018). Phytopathogen-induced changes to plant methylomes. Plant Cell Rep.

[24] Ma Y, Min L, Wang M, Wang C, Zhao Y, Li Y (2018). Disrupted genome methylation in response to high temperature has distinct affects on microspore abortion and anther indehiscence. Plant Cell.

[25] Wang C, Wang C, Zou J, Yang Y, Li Z, Zhu S (2019). Epigenetics in the plant-virus interaction. Plant Cell Rep.

[26] Kinoshita T, Seki M (2014). Epigenetic memory for stress response and adaptation in plants. Plant Cell Physiol.

[27] Thiebaut F, Hemerly AS, Ferreira PCG (2019). A role for epigenetic regulation in the adaptation and stress responses of non-model plants. Front Plant Sci.

[28] Vilchez JI, Yang Y, He D, Zi H, Peng L, Lv S (2020). DNA demethylases are required for myo-inositol-mediated mutualism between plants and beneficial rhizobacteria. Nat Plants.

[29] Wilkinson SW, Ton J (2020). Methylation moulds microbiomes. Nat Plants.

[30] Williams BP, Gehring M (2017). Stable transgenerational epigenetic inheritance requires a DNA methylation-sensing circuit. Nat Commun.

[31] Sudan J, Raina M, Singh R (2018). Plant epigenetic mechanisms: role in abiotic stress and their generational heritability. 3. Biotech..

[32] Law JA, Jacobsen SE (2010). Establishing, maintaining and modifying DNA methylation patterns in plants and animals. Nat Rev Genet.

[33] Du J, Johnson LM, Jacobsen SE, Patel DJ (2015). DNA methylation pathways and their crosstalk with histone methylation. Nat Rev Mol Cell Biol.

[34] Li Y, Kumar S, Qian W (2018). Active DNA demethylation: mechanism and role in plant development. Plant Cell Rep.

[35] Zhang H, Lang Z, Zhu JK (2018). Dynamics and function of DNA methylation in plants. Nat Rev Mol Cell Biol.

[36] Xu X, Xu M, Zhao Q, Xia Y, Chen C, Shen Z (2018). Complete genome sequence of Cd (II)-resistant Arthrobacter sp. PGP41, a plant growth-promoting bacterium with potential in microbe-assisted phytoremediation. Curr Microbiol.

[37] Chen C, Zhang HX, Wang AG, Lu M, Shen ZG, Lian CL (2015). Phenotypic plasticity accounts for most of the variation in leaf manganese concentrations in Phytolacca americana growing in manganese-contaminated environments. Plant Soil.

[38] Zhang CC, Yuan WY, Zhang QF (2012). RPL1, a gene involved in epigenetic processes regulates phenotypic plasticity in rice. Mol Plant.

[39] Kooke R, Johannes F, Wardenaar R, Becker F, Etcheverry M, Colot V (2015). Epigenetic basis of morphological variation and phenotypic plasticity in Arabidopsis thaliana. Plant Cell.

[40] Smirnoff N, Arnaud D (2019). Hydrogen peroxide metabolism and functions in plants. New Phytol.

[41] Gamir J, Pastor V, Sanchez-Bel P, Agut B, Mateu D, Garcia-Andrade J (2018). Starch degradation, abscisic acid and vesicular trafficking are important elements in callose priming by indole-3-carboxylic acid in response to Plectosphaerella cucumerina infection. Plant J.

[42] Bacete L, Melida H, Miedes E, Molina A (2018). Plant cell wall-mediated immunity: cell wall changes trigger disease resistance responses. Plant J.

[43] Lu Y, Yao J (2018). Chloroplasts at the crossroad of photosynthesis, pathogen infection and plant defense. Int J Mol Sci.

[44] Mauch-Mani B, Baccelli I, Luna E, Flors V (2017). Defense priming: an adaptive part of induced resistance. Annu Rev Plant Biol.

[45] Xu X, Zarecki R, Medina S, Ofaim S, Liu X, Chen C (2019). Modeling microbial communities from atrazine contaminated soils promotes the development of biostimulation solutions. ISME J.

[46] Lawson CE, Harcombe WR, Hatzenpichler R, Lindemann SR, Loffler FE, O’Malley MA (2019). Common principles and best practices for engineering microbiomes. Nat Rev Microbiol.

[47] Paredes SH, Lebeis SL (2016). Giving back to the community: microbial mechanisms of plant-soil interactions. Funct Ecol.

[48] Franco CMM, Gupta VVSR, Sharma AK (2021). Inoculation effects in the rhizosphere: diversity and function. Rhizosphere biology: interactions between microbes and plants.

[49] Trivedi P, Leach JE, Tringe SG, Sa T, Singh BK (2020). Plant–microbiome interactions: from community assembly to plant health. Nat Rev Microbiol.

[50] Pavet V, Quintero C, Cecchini NM, Rosa AL, Alvarez ME (2006). Arabidopsis displays centromeric DNA hypomethylation and cytological alterations of heterochromatin upon attack by Pseudomonas syringae. Mol Plant-Microbe Interact.

[51] Bewick AJ, Schmitz RJ (2017). Gene body DNA methylation in plants. Curr Opin Plant Biol.

[52] Zhang X, Yazaki J, Sundaresan A, Cokus S, Chan SW, Chen H (2006). Genome-wide high-resolution mapping and functional analysis of DNA methylation in arabidopsis. Cell..

[53] Edgar RC, Haas BJ, Clemente JC, Quince C, Knight R (2011). UCHIME improves sensitivity and speed of chimera detection. Bioinformatics..

[54] Edgar RC (2013). UPARSE: highly accurate OTU sequences from microbial amplicon reads. Nat Methods.

[55] Caporaso JG, Lauber CL, Walters WA, Berg-Lyons D, Lozupone CA, Turnbaugh PJ (2011). Global patterns of 16S rRNA diversity at a depth of millions of sequences per sample. Proc Natl Acad Sci U S A.

[56] Caporaso JG, Kuczynski J, Stombaugh J, Bittinger K, Bushman FD, Costello EK (2010). QIIME allows analysis of high-throughput community sequencing data. Nat Methods.

[57] Bolger AM, Lohse M, Usadel B (2014). Trimmomatic: a flexible trimmer for Illumina sequence data. Bioinformatics..

[58] Ghosh S, Chan CK (2016). Analysis of RNA-Seq data using TopHat and Cufflinks. Methods Mol Biol.

[59] Haas BJ, Papanicolaou A, Yassour M, Grabherr M, Blood PD, Bowden J (2013). De novo transcript sequence reconstruction from RNA-seq using the Trinity platform for reference generation and analysis. Nat Protoc.

[60] Li B, Dewey CN (2011). RSEM: accurate transcript quantification from RNA-Seq data with or without a reference genome. BMC Bioinformatics.

[61] Xi Y, Li W (2009). BSMAP: whole genome bisulfite sequence MAPping program. BMC Bioinformatics.

[62] Lister R, O’Malley RC, Tonti-Filippini J, Gregory BD, Berry CC, Millar AH (2008). Highly integrated single-base resolution maps of the epigenome in Arabidopsis. Cell..

[63] Schmittgen TD, Livak KJ (2008). Analyzing real-time PCR data by the comparative C(T) method. Nat Protoc.

[64] Dixon P (2003). VEGAN, a package of R functions for community ecology. J Veg Sci.

[65] Segata N, Izard J, Waldron L, Gevers D, Miropolsky L, Garrett WS (2011). Metagenomic biomarker discovery and explanation. Genome Biol.

[66] Love MI, Huber W, Anders S (2014). Moderated estimation of fold change and dispersion for RNA-seq data with DESeq2. Genome Biol.

[67] Breiman L (2001). Random Forests. Mach Learn.

[68] Deng Y, Jiang YH, Yang Y, He Z, Luo F, Zhou J (2012). Molecular ecological network analyses. BMC Bioinformatics.

[69] Langfelder P, Horvath S (2008). WGCNA: an R package for weighted correlation network analysis. BMC Bioinformatics.

