# Supplementary material for: Long-term effect of epigenetic modification in plant–microbe interactions: modification of DNA methylation induced by plant growth-promoting bacteria mediates promotion process
Source: Microbiome. 2022 Feb 24;10:36. doi: 10.1186/s40168-022-01236-9 (PMC8876431; doi:10.1186/s40168-022-01236-9)
Supplement: Supplementary file 2 — Additional file 1: Fig. S1. Schematic representation of the experimental design. Fig. S2. Differential abundances of bacterial communities in inoculated and non-inoculated soils at Day 3 and Day 30. Fig. S3. Co-occurrence networks of PGP5 over time, as affected by inoculation. Fig. S4. Variation in transcript profiles between the early and late phase. Fig. S5. Heatmap based on relative transcript abundances of genes involved in maintaining DNA methylation. Fig. S6. Overview of DNA methylation levels and differences in DNA methylation among samples. Fig. S7. Comparisons of abundances of strain PGP5 and PGP41 between inoculated and non-inoculated rhizosphere soils and roots by qPCR. Fig. S8. Detection of strain PGP41 in rhizosphere soils by FISH. Fig. S9. Detection of strain PGP5 in rhizosphere soils by FISH. Fig. S10. Detection of strain PGP41 in rhizosphere soils and roots with a GFP-tagged strain. Fig. S11. Detection of strains PGP41 and PGP5 in roots by FISH. Fig. S12. Detection of strains PGP41 and PGP5 in roots by 16S rRNA gene amplification. Fig. S13. Analysis of the effectiveness of soil sterilization. Fig. S14. Comparison of inoculum-induced growth promotion of P. americana in sterilized soils vs. unsterilized soils. Fig. S15. Inoculation induced P. americana growth promotion in sterilized soils were disrupted by DNA methylation inhibitor. Fig. S16. Effects of inoculation of strain PGP5 or PGP6 on contents of Fe, K, P and Mg in P. americana. Fig. S17. Effects of inoculation of strain PGP5 or PGP41 on height, fresh weight of stems and leaves, number of inflorescences, and fresh weight of inflorescences of P. americana. Fig. S18. Neighbor-joining tree based on 16S rRNA gene sequences showing the position of strain PGP41 within the genus Bacillus. Fig. S19. Variation in the rhizosphere microbiome between Day 3 and Day 30 at the functional level. Table S1. GO enrichment analyses of PGP5 DEGs. Table S2. GO enrichment analyses of PGP41 DEGs. Table S3. Primers used [file 40168_2022_1236_MOESM2_ESM.docx]

**Supplementary Text**

**1. Isolation of strain PGP5**

The strain PGP5 used in the present study was isolated from the rhizosphere soils of *Phytolacca americana* in Fang County, Hubei province, China (33°34′N, 107°34′W). Phylogenetic analysis based on 16S rRNA gene sequences showed that the strain PGP5 (MH087460) was clustered together with *Bacillus megaterium* and *B. aryabhattai* (Fig. S18). The similarity between 16S rRNA gene sequences of PGP5 and *B. megaterium* DN3 (MK307786.1) was 100%. The result suggested that strain PGP5 could be assigned to the genus *Bacillus*.

**2. Construction of sequencing libraries**

For 16S rRNA amplicon sequencing, Total DNA from 500 mg soil was extracted using the PowerSoil DNA Isolation Kit (MoBio, Carlsbad, CA, USA). The purity and concentration of the extracted DNA were measured using a NanoDrop spectrophotometer (ND2000, Thermo Fisher Scientific, Wilmington, DE, USA). The conserved bacteria-specific primer set 341F/806R [70] was used to amplify the V4 region of the 16S rRNA gene, as described previously [45]. Amplicon sequencing was performed on an Illumina MiSeq platform according to the standard protocols at Biozeron Biotechnology Co. (Shanghai, China).

For RNA sequencing, total RNA was extracted from root tissues using TRIzol reagent according the manufacturer’s instructions (Invitrogen, Carlsbad, CA, USA), then genomic DNA was removed using DNase I (Takara Bio, Shiga, Japan). RNA quality was analyzed with an Agilent 2100 Bioanalyzer and quantified using a Nanodrop 2000. RNA-seq libraries were constructed using the Illumina TruSeq RNA Sample Preparation Kit (San Diego, CA, USA) following standard procedures. The libraries were sequenced on the Illumina HiSeq 2500 system at Biozeron Biotechnology Co. Two libraries were excluded from the analyses based on sequencing quality and reproducibility with the other replicates.

For the methylome analyses, total DNA was extracted from roots using a Plant DNA Kit (Omega Bio-Tek, Norcross, GA, USA). The bisulfite conversion of DNA was carried out using an EZ DNA Methylation-Gold Kit (Zymo Research, Irvine, CA, USA). The TruSeq DNA methylation kit was used to construct Illumina sequencing libraries following the manufacturer’s instructions. Lambda-DNA was used as a control. Paired-end sequencing was carried out on the Illumina HiSeq 2500 platform at Biozeron Biotechnology Co.

**3. Comparison of rhizosphere microbiomes between early and late phase.**

At taxonomic-level, there were much fewer differentially abundant genera at Day 3 among treatments (CK vs. PGP5/41) than those among times (Day 3 vs. Day 30) (Fig. 2a-c), consistent with the conclusion that rhizosphere bacteria are mainly influenced by plant development.

At functional-level, we identified 1671 COGs with differential abundance in the CK-Day 3 vs. CK-Day 30 comparison; the number of these differential abundant COGs was 21-fold more than those of differential abundant COGs identified in the CK-Day 3 vs PGP5-Day 3 or PGP41-Day 3 comparison (Fig. S19). For these COGs, all Day 3 samples or Day 30 samples showed similar abundance patterns (Fig. S19). These results are consistent with the above conclusion that the rhizosphere microbiome is mainly influenced by plant growth, which may attenuate or eliminate the early effects of inoculations.

**4. qPCR for 16S rRNA gene copy number determination**

The 16S rRNA gene was amplified by PGP5- or PGP41-specific primers. The primers used are listed in Table S3. The PCR product was purified and then cloned into a pMD^TM^19-T vector. The resulting plasmids containing 16S rDNA sequence of PGP5 or PGP41 were selected. The standard curves were constructed with 10-fold serial dilutions of the pMD^TM^19-T, ranging from 4.39×10^2^ to 4.39×10^9^ (PGP5) and 1.96×10^3^ to 1.96×10^9^ (PGP41) copies/μL for strain PGP5 and PGP41 respectively. The concentration of the plasmids was measured using a NanoDrop ND-2000 spectrophotometer (ND2000, Thermo Scientific, DE, USA), and the corresponding copy number of 16S rRNA gene was calculated using the following equation [71]:

$$16S rRNA gene (copy)=\frac{6.02 \times{10}^{23} (copy/mol) \times plasmid amount (g/\mu L)}{plasmid length \left( \mathrm{bp} \right)\times660 (g/mol/bp)}$$

The Ct values in each dilution were measured in duplicate using a qPCR. To generate the standard curves, the Ct values were plotted against the logarithm of their copy numbers. Each standard curve was generated by a linear regression of the plotted points. The qPCR was performed on an ABI StepOnePlus real-time PCR system with TB Green Premix Ex Taq (Tli RNaseH Plus) kit (Takara Bio).

**5. Fluorescence in situ hybridization (FISH)**

To prepare soil samples for FISH, the fresh soil was weighed and diluted with phosphate-buffered saline (PBS) (7 mM Na_2_HPO_4_, 3 mM NaH_2_PO_4_, 130 mM NaCl; pH 7.2), and coated evenly on a glass slide. For fixation, the paraformaldehyde solution (4% final concentration) was added, and incubated for 20 min at room temperature. For cells in liquid culture, the cultures were centrifuged at 5000 rpm for 5 min, and the cells were suspended with 500 μL PBS. The cells were coated evenly on a glass slide and then fixed as described above. After fixation, the cells were washed with PBS for 5 min and repeated for 3 times. For root samples, the fresh roots were fixed in 4% paraformaldehyde solution for 2-12 h. The fixative was removed by washing the roots with PBS. The roots were air dried, and dehydrated by successive 3-min incubations in 50, 80, and 100% ethanol. The dried and dehydrated root material preparations were embedded in paraffin. Paraffin sections were cut from root specimens, dewaxed twice for 15 min each in xylene, and rehydrated through an ethanol series (100, 85, 75, and 0 % ethanol for 5 min). Then sections were permeabilized with 20 µg/mL proteinase K for 15 min at 37 °C, and then washed with PBS for 3 times, and subsequently air-dried prior to hybridization.

The PGP5- and PGP41-targeted oligonucleotide probes (Table S3) labeled with carbocyanine dye CY3 were used. The root materials were hybridized with the CY3-labeled oligonucleotide probe (5 ng/µL) at 37°C for one night. Following hybridization, the slides were washed as follows: once in 2 × SSC solution (750 mM NaCl and 75 mM Na citrate, pH 7.0) at 37°C for 10 min; twice in 1 × SSC at 37°C for 5 min; and once in 0.5 × SSC at room temperature for 10 min. The sections were then stained with 4′6′-diamidino-2-phenylindole (DAPI, 2 μg/mL). Images were acquired with a Nikon Eclipse Ci upright microscope.

**6. Construction of GFP-tagged strain PGP41 (PGP41-*gfp*)**

**6.1. Construction of recombinant plasmid pSC-GFP**

The plasmid pSC123 was preserved in our laboratory and had resistance to chloramphenicol and kanamycin. *KpnI* and *SalI* sites on the plasmid were selected for digestion with corresponding restriction nuclease. The plasmid was digested by double enzymes to obtain linear fragment. Then, the *gfp* reporter gene (encoding green fluorescent protein) was amplified by upstream primers (5’-TCCACTAGTTCTAGAGTCGACGAAATGAGCTGTTGACAATTAATCATC-3’, the homologous sequence site is underlined) and downstream primers (5’-CCTCGACGCGTCCTCGGTACCTGTAACACTGGCAGAGCATTACG-3’, the homologous sequence site is underlined). After PCR amplification, the target fragment and linear vector were purified and ligated together and then transformed into *Escherichia coli* DH5α. The recombinant plasmid pSC-GFP was then obtained by plasmid extraction kit (Vazyme, Najing, China).

**6.2. Bacterial transformation by electroporation**

The recipient bacteria were cultured for 16 h in LB broth (1% tryptone, 0.5% yeast extract, and 0.5% NaCl) at 37°C and 0.5 mL of the culture was inoculated into 100 mL fresh LB broth. The culture (OD_600_ = 1.0) was centrifuged at 4000 rpm at 4°C for 20 min, and the cells were suspended with pre-cooled 10% glycerol and then incubated on ice for 10 min. The cells were then centrifuged at 4000 rpm at 4°C for 10 min, washed with electroperforation buffer (25% PEG6000 and 0.1 M sorbitol) for 3 times, and suspended in 2 mL of the same electroperforation buffer. A total of 0.1 mL of the competent cells was mixed with plasmid DNA (400 ng) in a 0.1 cm gap electric rotary cup, which was stored in ice for 5 min. Then, a single electric pulse (2200 V, 400 Ω, 25 μF) was applied to the rotary cup using a rotary meter. After electroporation, 1 mL of LB broth containing 1 M sorbitol was added immediately. The mixture were then incubated at 37°C for 3 h under shaking conditions, and 0.1 mL of the mixture were plated onto LB agar medium containing 50 μg/mL kanamycin. After incubation at 37°C for 16 h, the transformants were selected for verification.

**References**

45. Xu X, Zarecki R, Medina S, Ofaim S, Liu X, Chen C, et al. Modeling microbial communities from atrazine contaminated soils promotes the development of biostimulation solutions. ISME J. 2019;13:494–508.

70. Mori H, Maruyama F, Kato H, Toyoda A, Dozono A, Ohtsubo Y, et al. Design and experimental application of a novel non-degenerate universal primer set that amplifies prokaryotic 16S rRNA genes with a low possibility to amplify eukaryotic rRNA genes. DNA Res. 2014;21:217–227.

71. Lee C, Kim J, Shin SG, Hwang S. Absolute and relative QPCR quantification of plasmid copy number in Escherichia coli. J Biotechnol. 2006;123:273–280.

**Supplementary Figures**


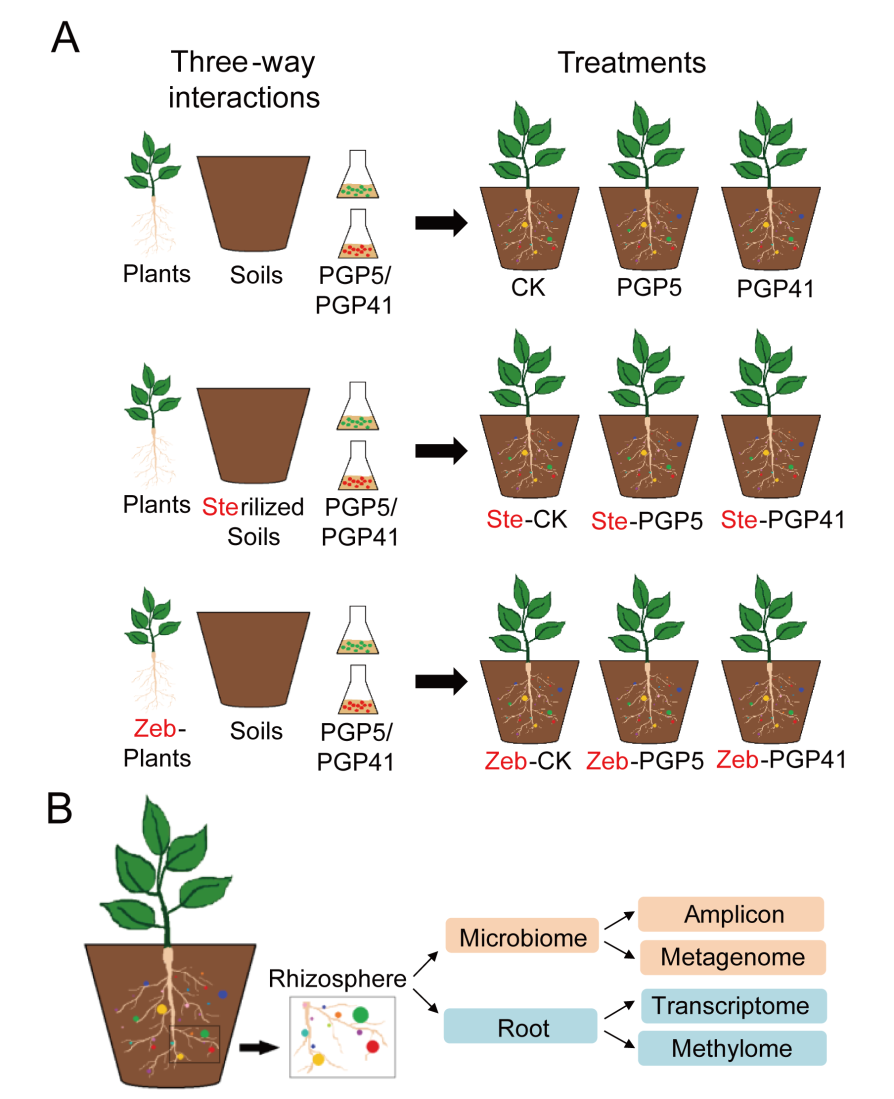


**Fig. S1**. Schematic representation of the experimental design. (A) Both control and zebularine (Zeb)-treated *P*. *americana* were transferred to bulk soils, sterilized (Ste)-soils, or soils with inoculum (strain PGP5 or PGP41), and harvested at 0, 3, 7, 15, 21, and 30 days after transplantation. (B) The complex interactions in the rhizosphere were comprehensively studied by a multi-omics approach. Variation in the rhizosphere microbiome at taxonomic and functional levels was analyzed by amplicon and metagenome sequencing, respectively. Changes in rhizosphere roots at transcriptional and epigenetic levels were analyzed by transcriptome and methylome sequencing, respectively.


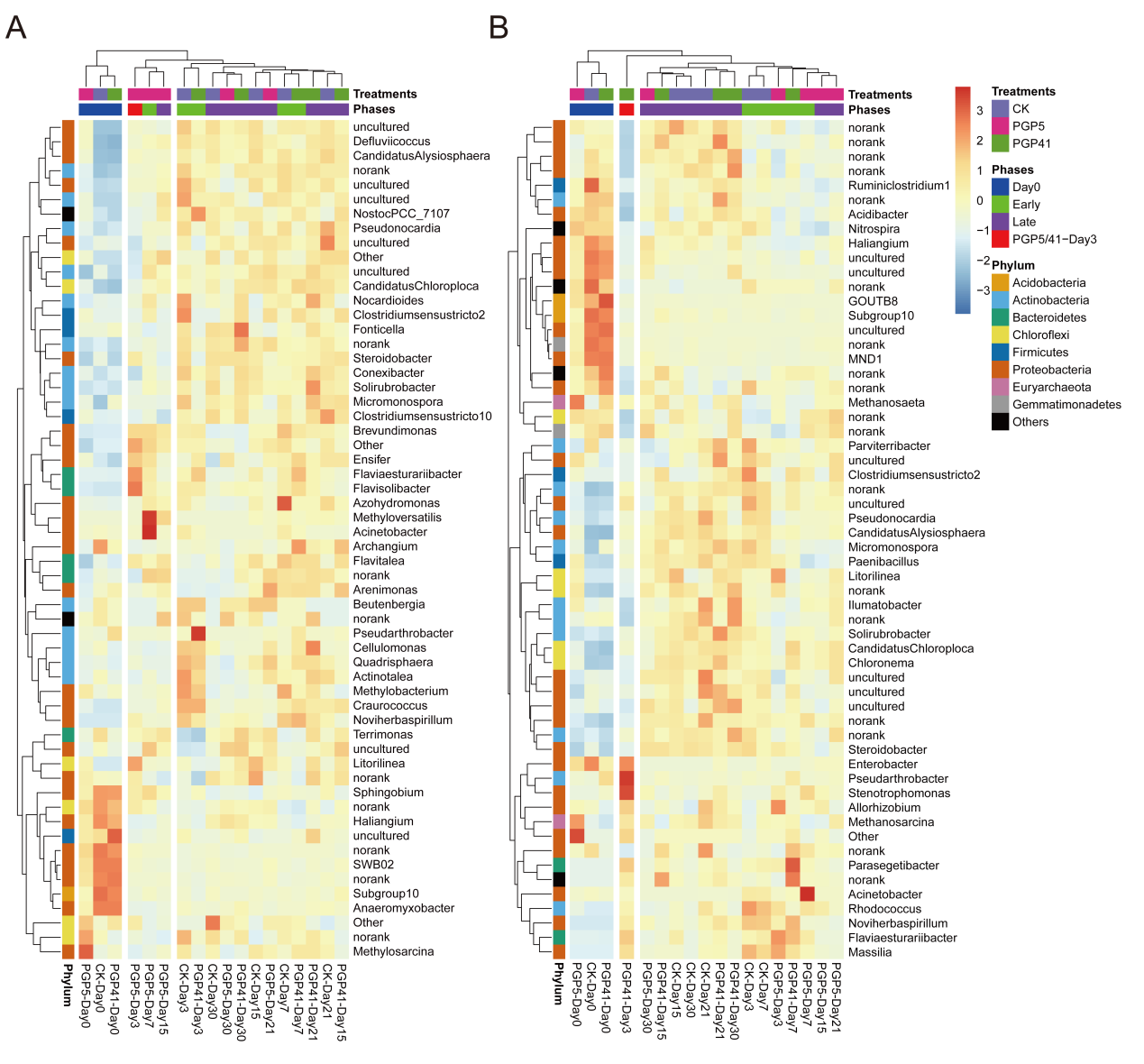


**Fig. S2**. Differential abundances of bacterial communities in inoculated and non-inoculated soils at Day 3 and Day 30. Heatmaps are based on relative abundances of enriched genera in comparisons of (A) PGP5-Day 3 vs. CK-Day 3 and (B) PGP41-Day 3 vs. CK-Day 3. The results show that the variation is inoculum-specific in the early phase (days 3–15 for PGP5 and day 3 for PGP41).


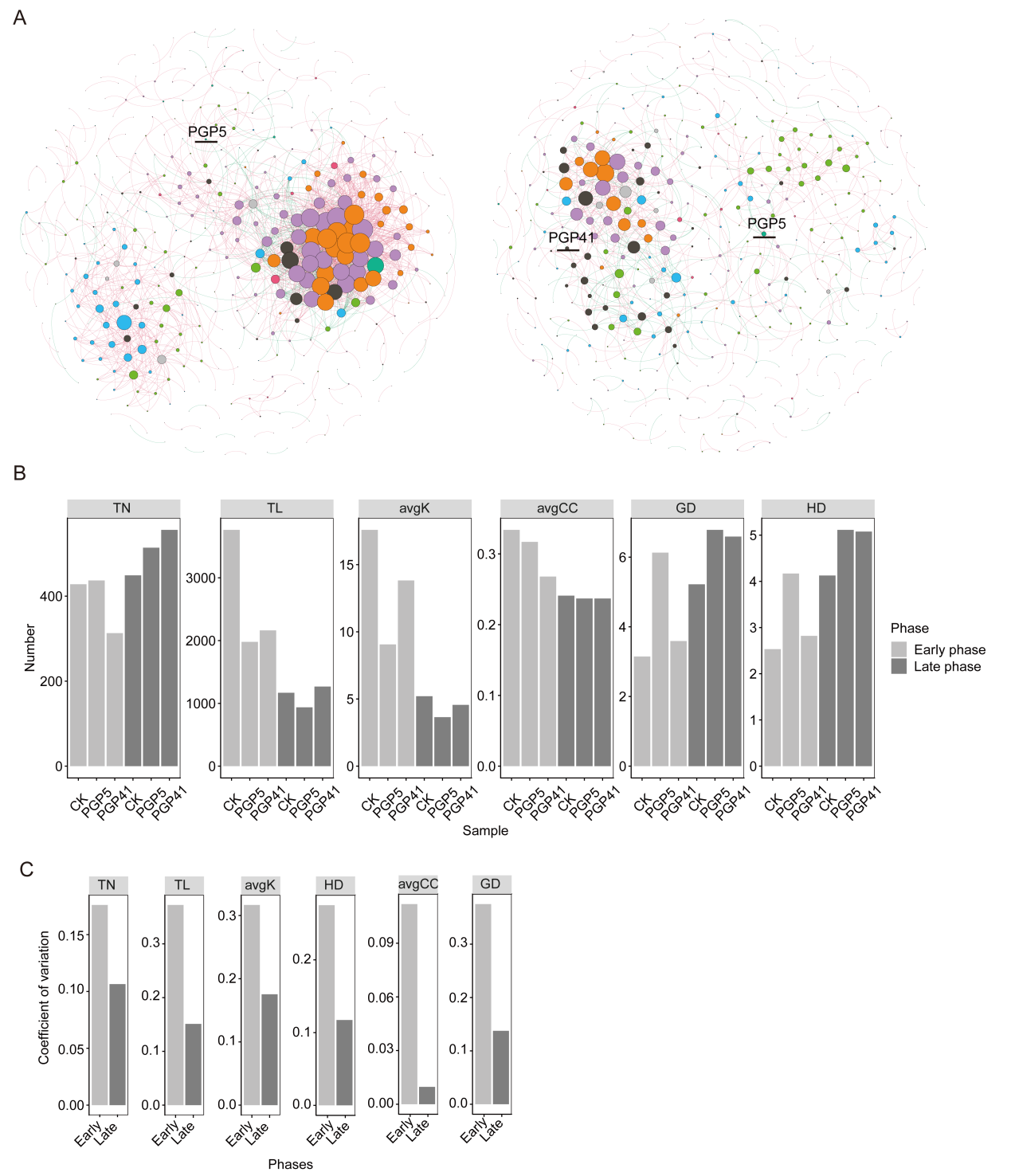


**Fig. S3**. Co-occurrence networks of PGP5 over time, as affected by inoculation. (A) Networks of the PGP5-inoculated microbiome in the early (left) and late (right) phases. Nodes refer to OTUs; edges refer to significant correlations. The color of each node indicates the phylum; the size of each node is proportional to the degree. The OTUs represent strains PGP5 and PGP41 (with 100% identity) are labeled in the networks. (B) Variation in global network properties among samples between early and late phases. (C) Coefficient of variation in global network properties (based on panel b) among CK, PGP5 and PGP41 samples in the late phase is much lower compared to those in early phase, showing much higher similarity between inoculated and control microbiomes at the late phase compared to the early phase. TN, total nodes; TL, total links; avgK, average degree; avgCC, average clustering coefficient; GD, average path length; HD, harmonic geodesic distance.


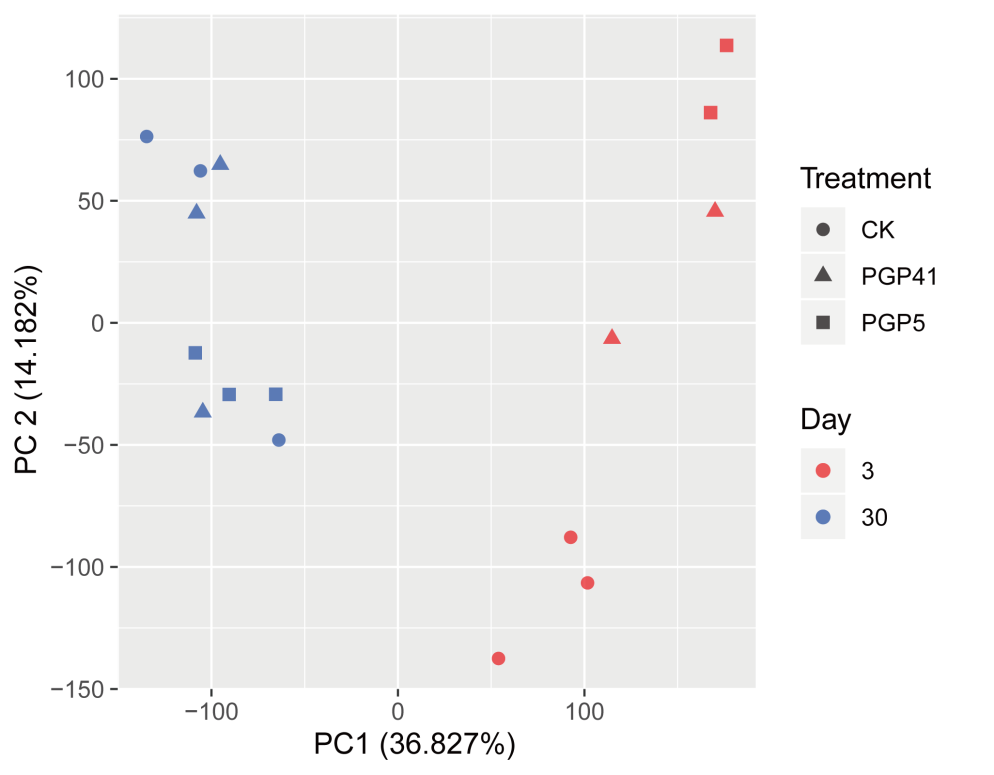


**Fig. S4**. Variation in transcript profiles between the early and late phase. PCA showing shifts of transcript profiles with plant development, and variation in the transcriptome induced by inoculation in the early phase, but not the late phase.


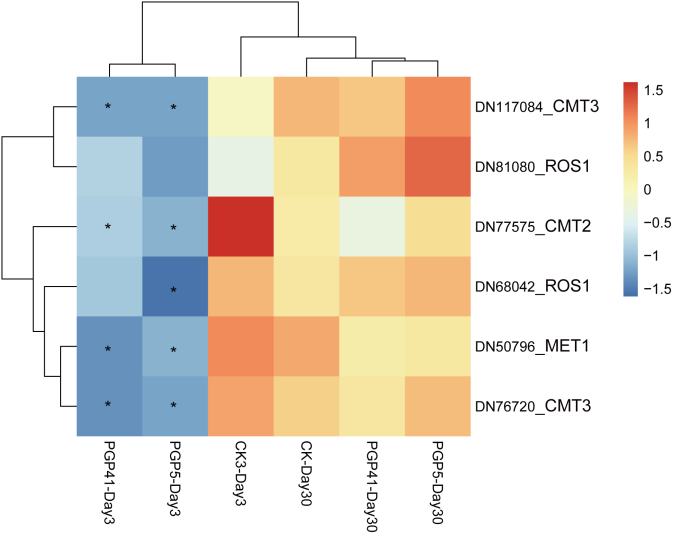


**Fig. S5**. Heatmap based on relative transcript abundances of genes involved in maintaining DNA methylation, showing significantly different expression patterns between inoculated and non-inoculated roots in the early phase but not the late phase. Red, increased transcript abundance; blue, decreased transcript abundance.


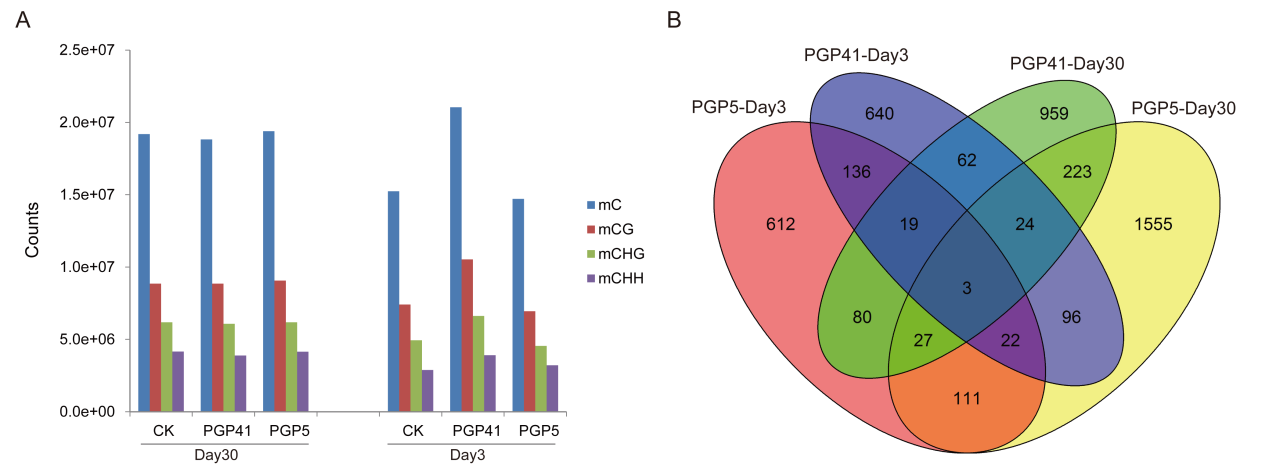


**Fig. S6**. Overview of DNA methylation levels and differences in DNA methylation among samples. (A) Number of methylated cytosines (mCs) in each sample. (B) Venn diagram of DMRs in inoculated samples overlapping between the early and late phases.


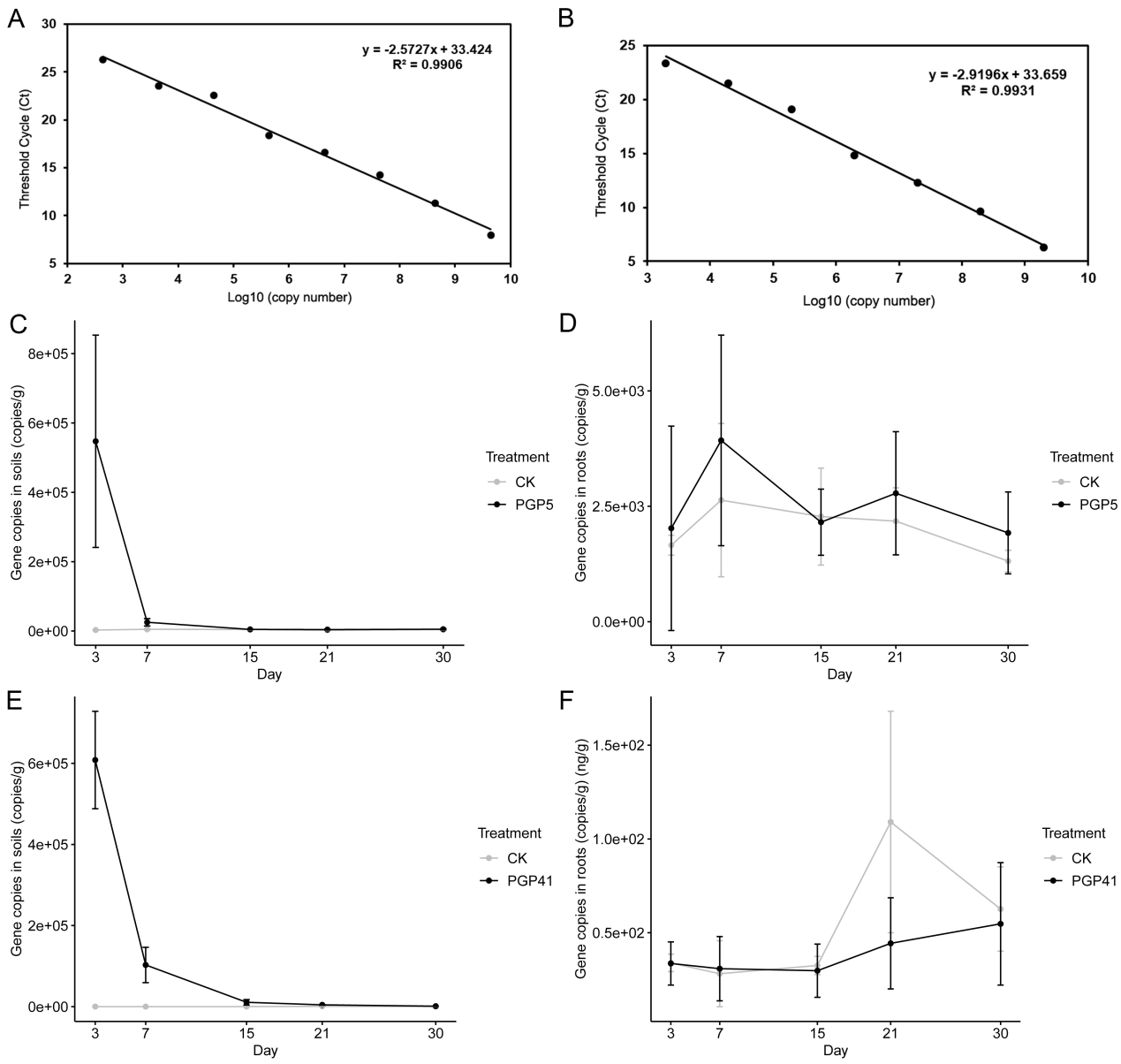


**Fig. S7.** Comparisons of abundances of strain PGP5 and PGP41 between inoculated and non-inoculated rhizosphere soils and roots by qPCR. A-B, Linear relationship between threshold cycles (Ct) and the input copy number of 16S rRNA gene from strain PGP5 (A) and PGP41 (B). C-D, Abundances of 16S rRNA gene of strain PGP5 in rhizosphere soils (C) and roots (D). CK, non-inoculated rhizosphere soils or roots; PGP5, inoculated rhizosphere soils or roots with strain PGP5. E-F, Abundances of 16S rRNA gene of strain PGP41 in rhizosphere soils (E) and roots (F). CK, non-inoculated rhizosphere soils or roots; PGP41, inoculated rhizosphere soils or roots with strain PGP41. The inoculation significantly increased the abundances of strain PGP5 and PGP41 in rhizosphere soils at early state, and the abundances of both inocula rapidly decreased to the same level in control soils. No significant differences were detected in inoculated roots compared to non-inoculated roots for both strain PGP5 and PGP41, indicating no colonization of inocula in roots. The primers used are listed in Table S3.


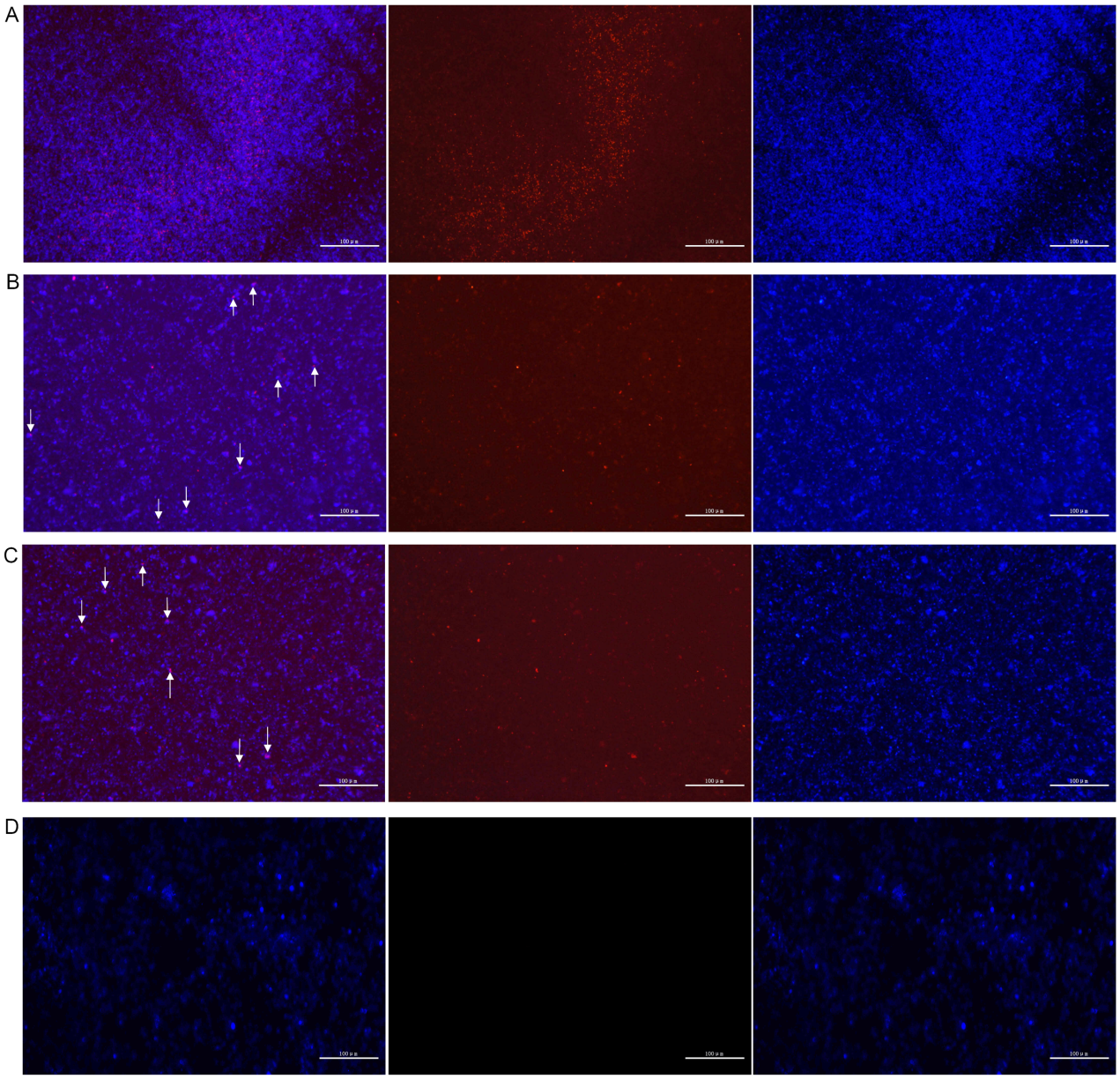


**Fig. S8.** Detection of strain PGP41 in rhizosphere soils by FISH. A, Images of strain PGP41 showing the strain was successfully labeled using a Cy3-labeled FISH probe (red fluorescence). Right, the DAPI stained bacterial cells; Middle, the fluorescence microscopy image showing the FISH signal; Left, the overlapped image. B, Images of rhizosphere soils which was taken immediately after inoculation and used as a positive control. C-D, Images of inoculated rhizosphere soils at Day 3 (C) and 30 (D). Some of the cells with a FISH signal are indicated by white arrows. The images reveal the presence of a FISH signal in rhizosphere soils at Day3 but absence at Day 30, suggesting the elimination of strain PGP41 from rhizosphere soils.


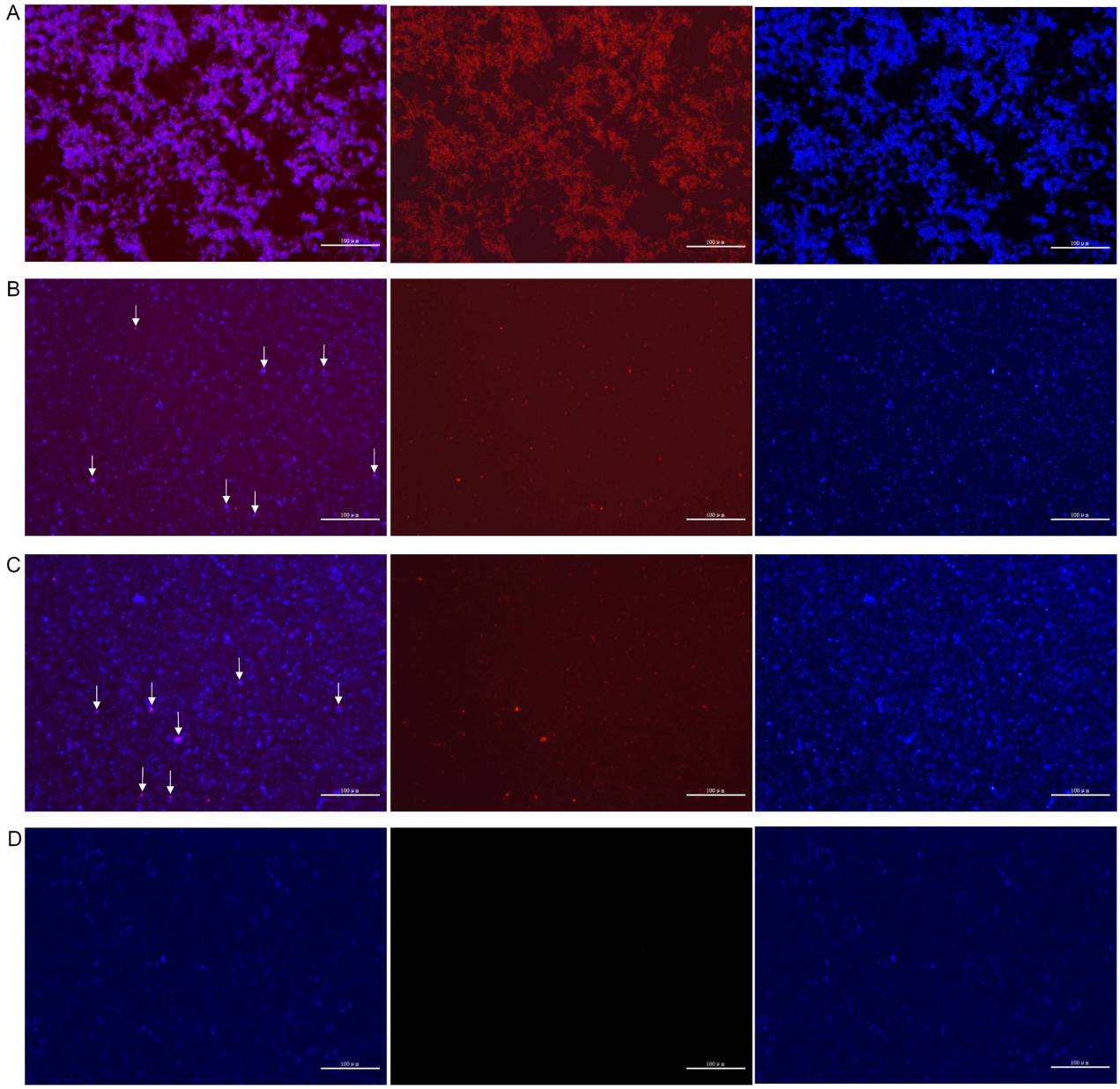


**Fig. S9.** Detection of strain PGP5 in rhizosphere soils by FISH. A, Images of strain PGP5 showing the strain was successfully labeled using a Cy3-labeled FISH probe (red fluorescence). Right, the DAPI stained bacterial cells; Middle, the fluorescence microscopy image showing the FISH signal; Left, the overlapped image. B, Images of rhizosphere soils which was taken immediately after inoculation and used as a positive control. C-D, Images of inoculated rhizosphere soils at Day 3 (C) and 30 (D). Some of the cells with a FISH signal are indicated by white arrows. The images reveal the presence of a FISH signal in rhizosphere soils at Day3 but absence at Day 30, suggesting the elimination of strain PGP5 from rhizosphere soils.


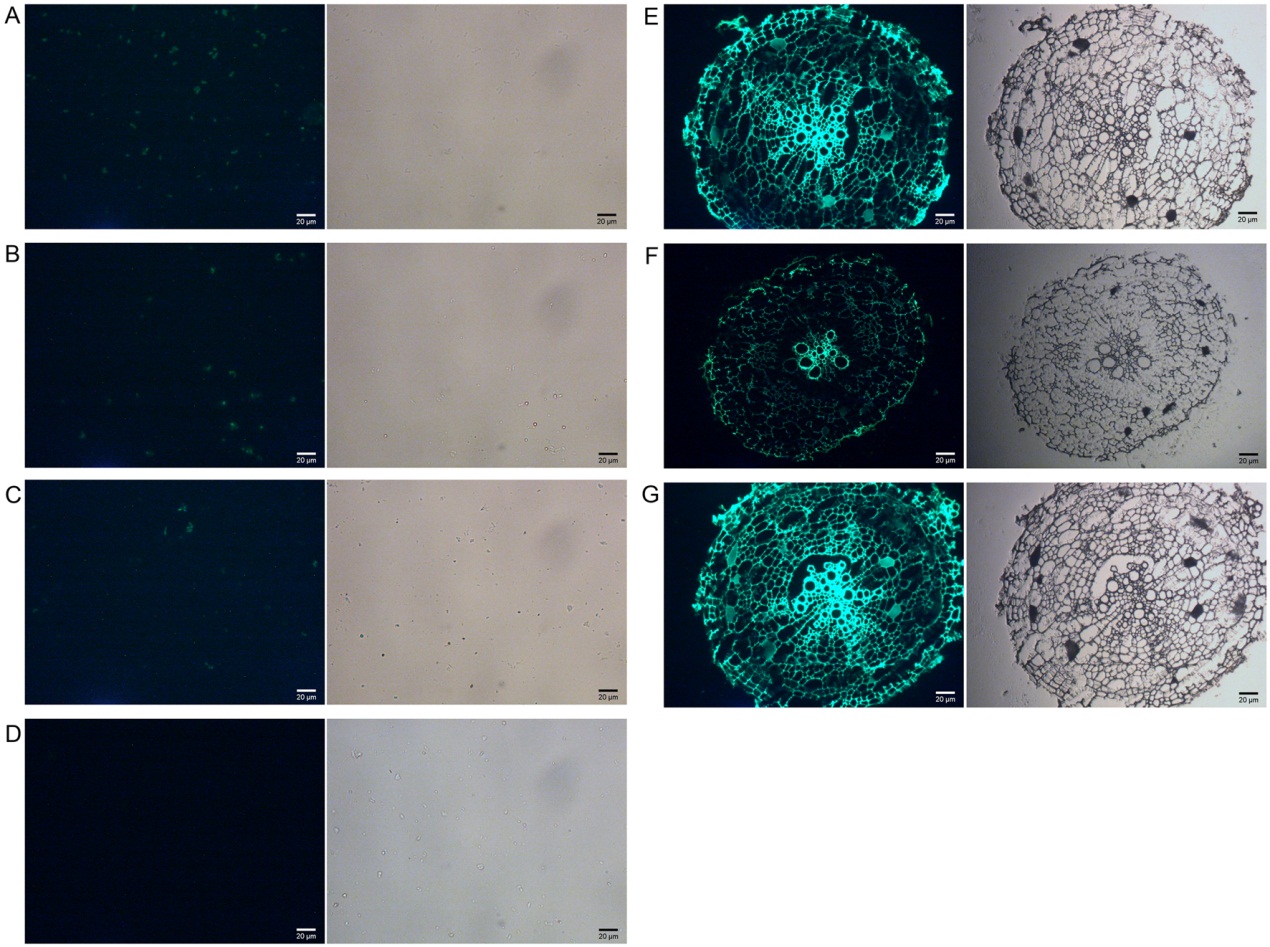


**Fig. S10.** Detection of strain PGP41 in rhizosphere soils and roots with a GFP-tagged strain. A, a GFP-tagged strain of PGP41 was successfully constructed. Light (right) and fluorescence (left) microscopy of the GFP-tagged strain are showed. B-D, Light (right) and fluorescence (left) microscopy of the GFP-tagged strain in inoculated rhizosphere soils at Day 3 (B), 7 (C), and 15 (D). E-G, Light (right) and fluorescence (left) microscopy of the GFP-tagged strain in inoculated roots at Day 3 (E), 7 (F), and 15 (G). The GFP-tagged strain was detected in rhizosphere soils at Day 3 and 7 but not at Day 15, suggesting that the present of strain PGP41 in the rhizosphere soils at early stage which was eliminated from the rhizosphere soils at late stage. For roots, no GFP-tagged strain was detected at Day 3, 7, or 15. The images suggest no colonization of strain PGP41 in roots of *P. americana*.


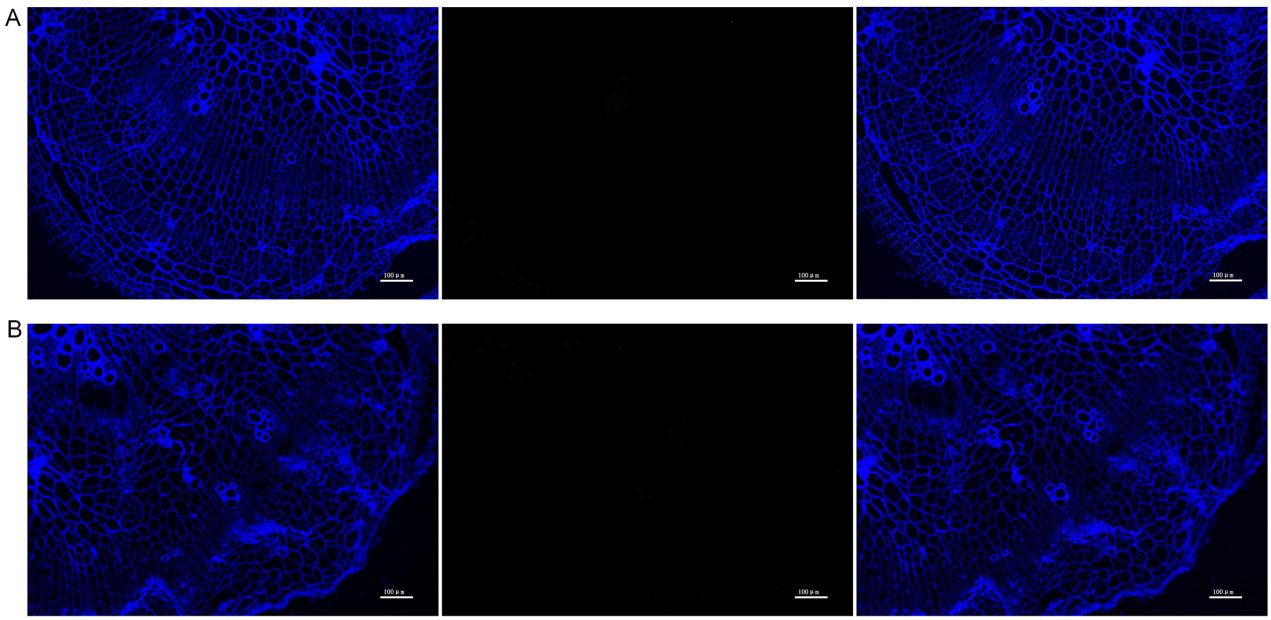


**Fig. S11.** Detection of strains PGP41 and PGP5 in roots by FISH. A, Images of horizontal slices of roots inoculated with strain PGP41 at Day 30. B, Images of horizontal slices of roots inoculated with strain PGP5 at Day 30. Right, the DAPI stained image; Middle, the fluorescence microscopy image; Left, the overlapped image. The root cells are visible via DAPI staining. The red fluorescence (labeled fluorescence, Middle) was not detected. The images reveal the absence of a FISH signal in roots inoculated with strain PGP41 or PGP5.


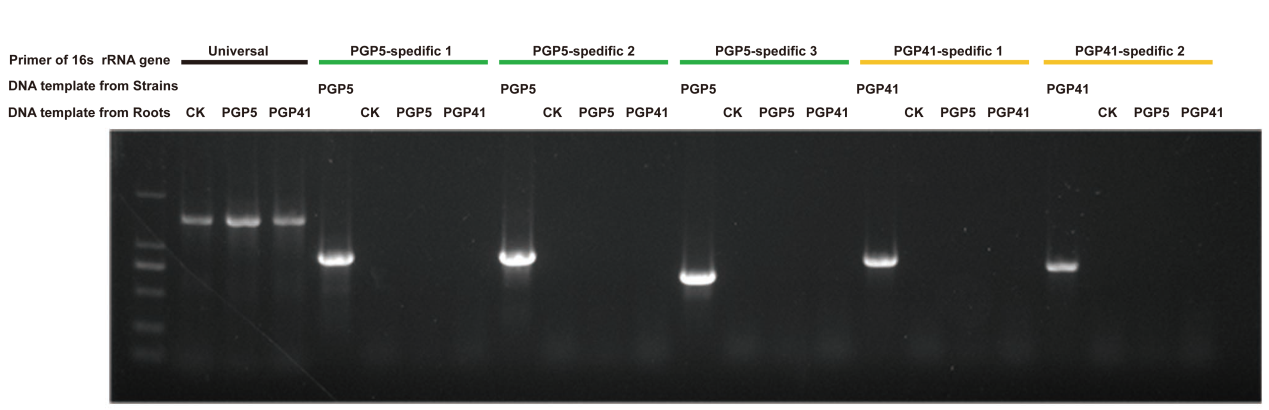


**Fig. S12**. Detection of strains PGP41 and PGP5 in roots by 16S rRNA gene amplification. The amplification was successful with PGP5- and PGP41-specific 16S rRNA gene primers from DNA of strain PGP5 and PGP41 respectively, but it was failed for root DNA. To exclude the possibility that bacterial DNA extracted from roots was too diluted to be detected, we used the universal bacterial 16S rRNA gene primers as negative control. The amplification from root DNA was successful using universal bacterial 16S rRNA gene primers, indicating that bacterial DNA was existed and detectable in root DNA but it was not from PGP5 or PGP41. The primers used are listed in Table S3.


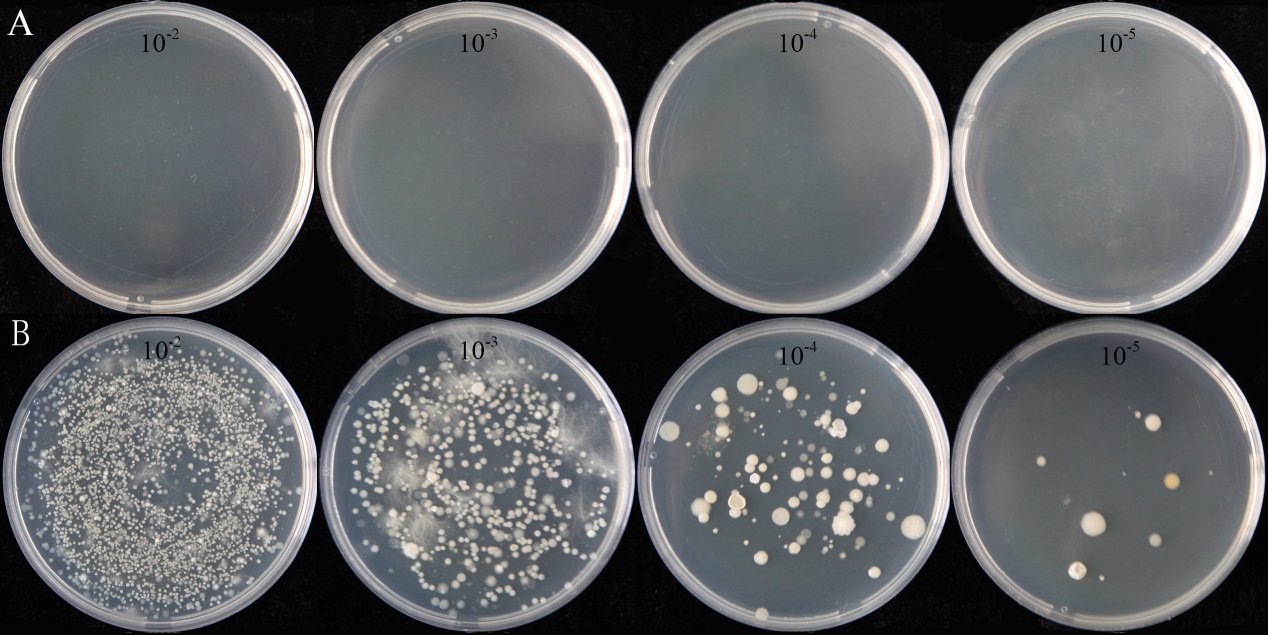


**Fig. S13.** Analysis of the effectiveness of soil sterilization. Microorganisms were isolated from sterilized (A) and unsterilized soils (B) by gradient dilution method. The dilution ratios are showed on the agar plates.


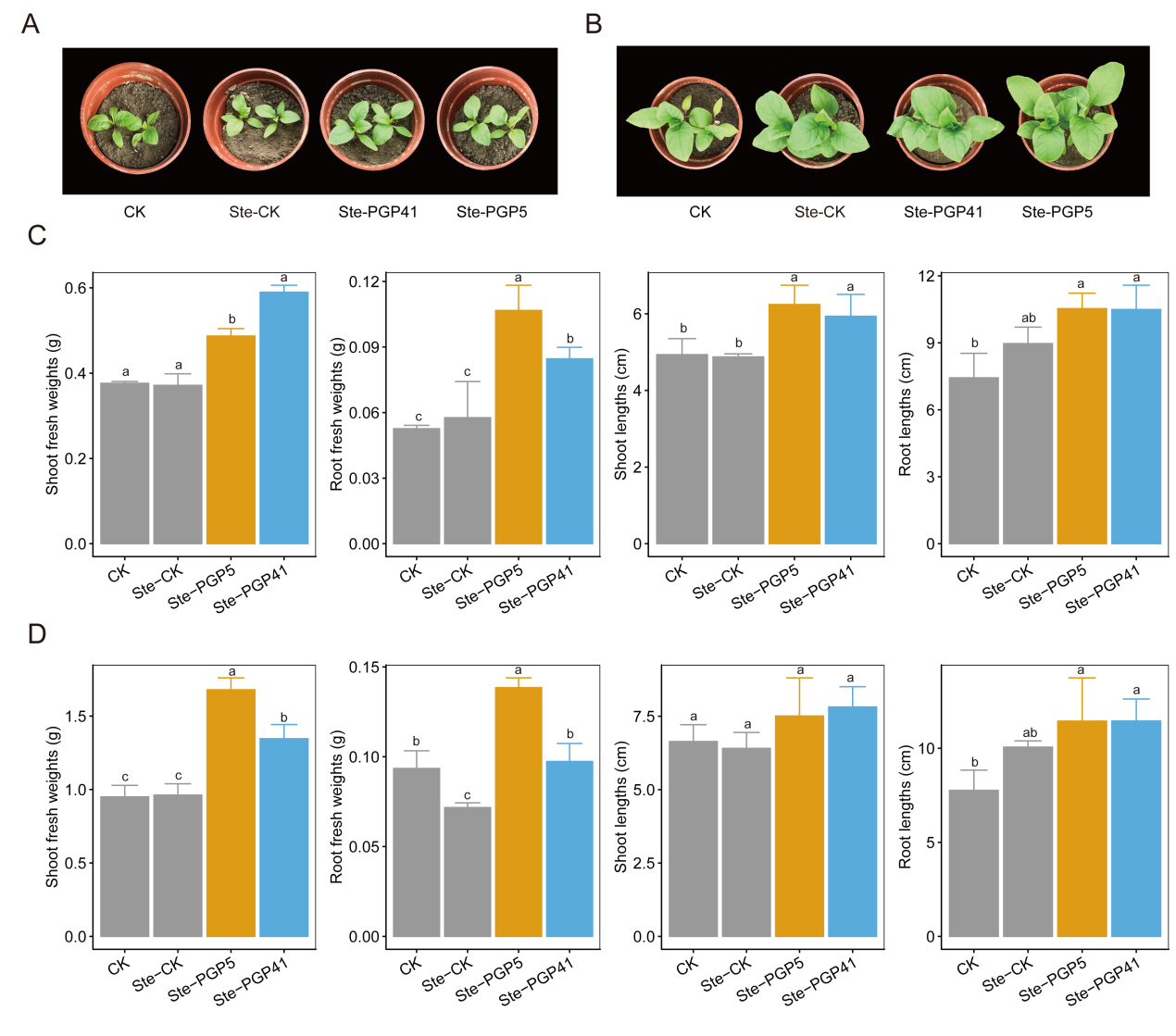


**Fig. S14**. Comparison of inoculum-induced growth promotion of *P*. *americana* in sterilized soils vs. unsterilized soils. (A, B) Aerial part of *P. americana* under different treatments at Day 3 (A) and Day 30 (B). CK, non-inoculated plants treated with unsterilized soils; Ste-CK, non-inoculated plants treated with sterilized soils; Ste-PGP5, PGP5-inoculated plants treated with sterilized soils; Ste-PGP41, PGP41-inoculated plants treated with sterilized soils. (C, D) The weights and lengths of shoots and roots of *P. americana* at Day 3 (c, n = 3) and Day 30 (d, n=3). Different letters indicate significant differences (Duncan’s test, P < 0.05). To test the effect of the seedling age when treated with inoculation on the inoculum-induced growth promotion, one month old seedlings (i.e. one month younger than seedlings used in other experiments) were used. The results showed that the inoculations induced growth promotion of *P*. *americana* in sterilized soils, and the seedling age with initial inoculation was flexible.


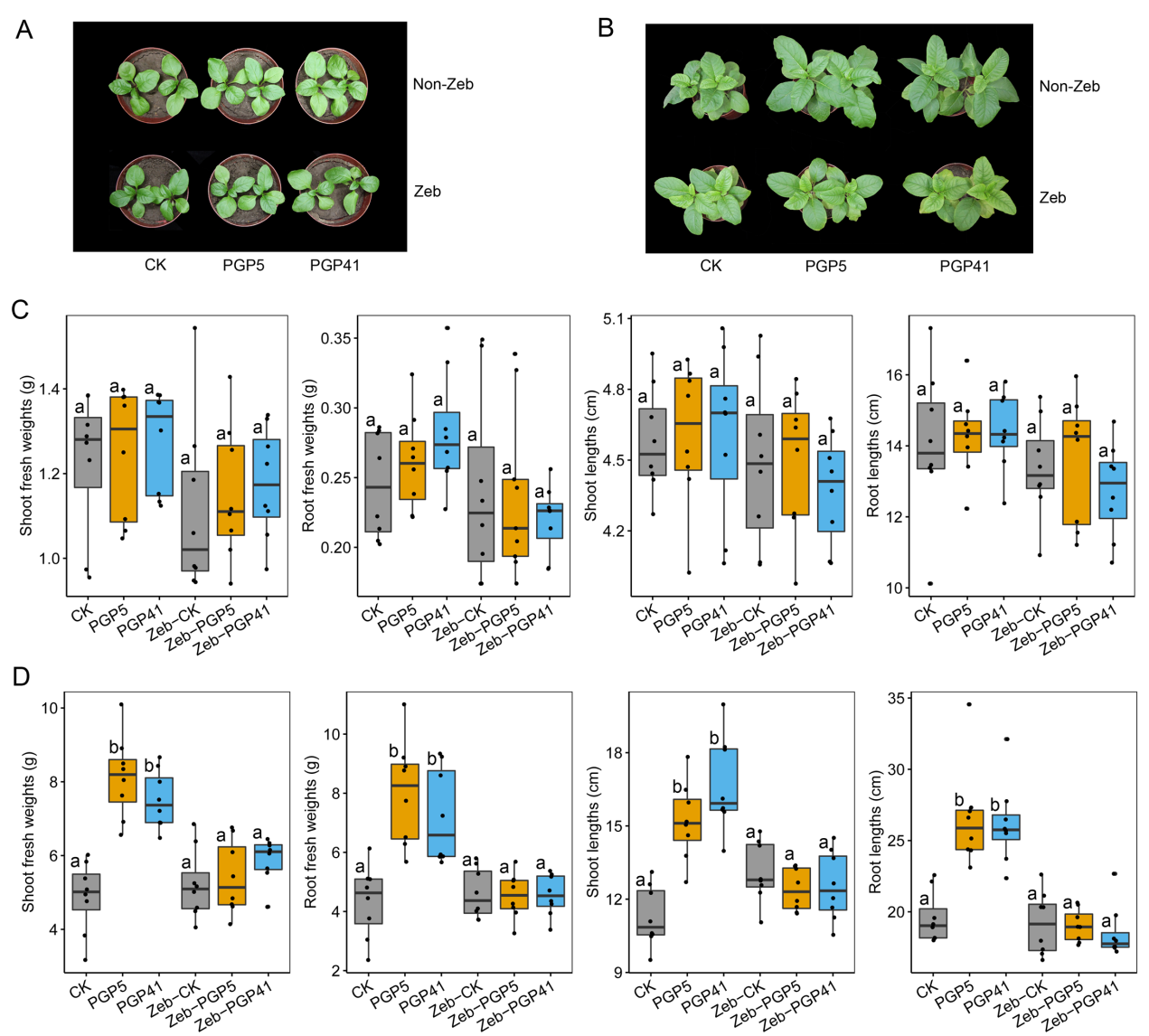


**Fig. S15.** Inoculation induced *P. americana* growth promotion in sterilized soils were disrupted by DNA methylation inhibitor. A-B, Aerial part of *P. americana* under different treatments at Day 3 (A) and Day 30 (B) in sterilized soils. The plants were treated with zebularine (Zeb), a DNA methylation inhibitor, to investigate the role of DNA methylation in the growth-promoting process. Zeb, Zeb-treated samples; Non–Zeb, samples without Zeb-treatment. C-D, Comparison of inoculation-induced *P. americana* growth promotion in sterilized soils with and without Zeb treatment at Day3 (C, n = 8) and Day30 (D, n = 8). The weights and lengths of both shoots and roots are shown. Different letters indicate significant differences (Duncan’s test, P < 0.05).


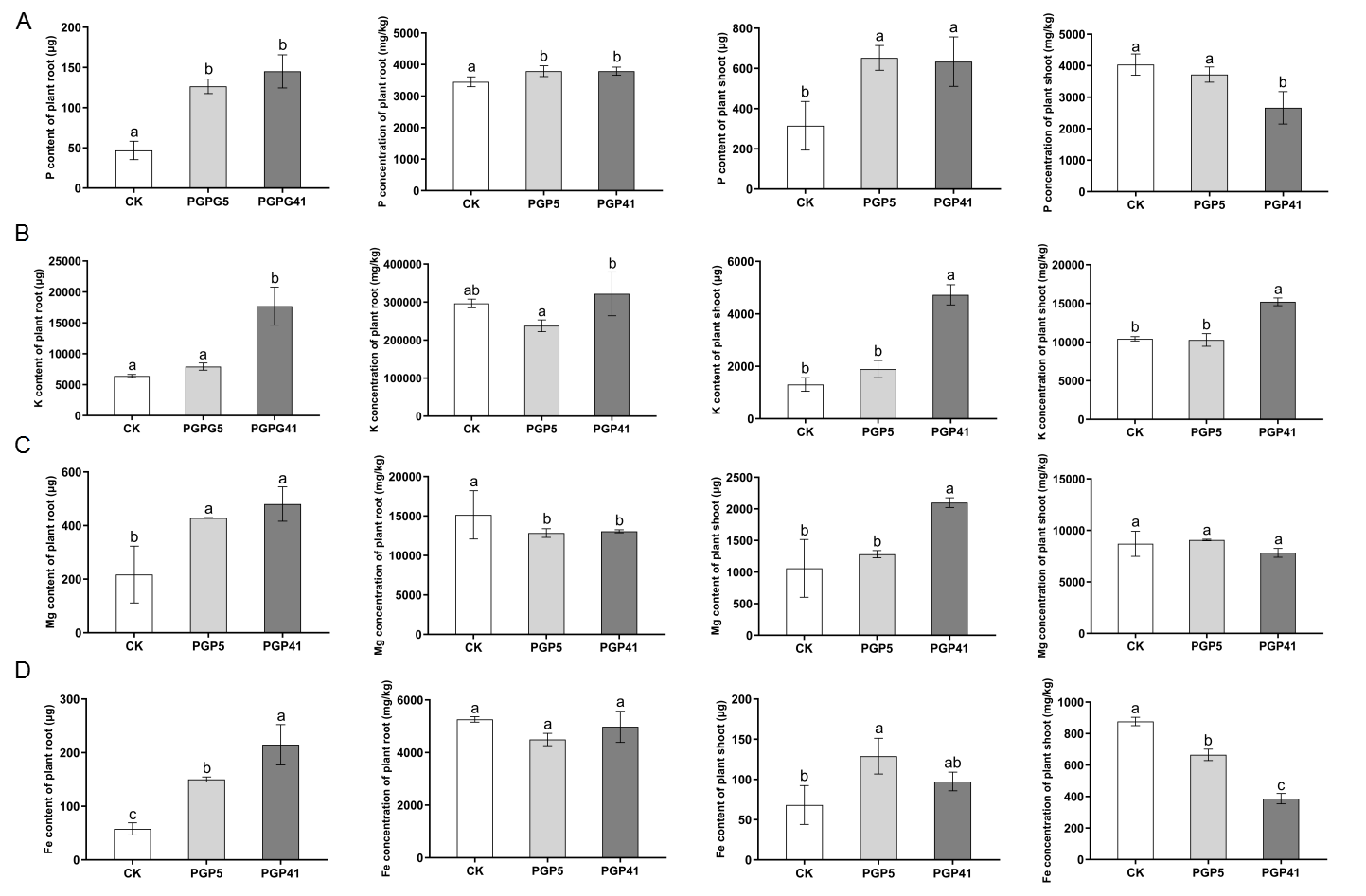


**Fig. S16.** Effects of inoculation of strain PGP5 or PGP6 on contents of P (A), K (B), Mg (C) and Fe (D) in *P. americana*. The total contents and contents per unit of mass (mg/kg) of these major and minor elements in roots and shoots of *P. americana* are shown. Values are means ± SD of three biological replicates and different lowercase letters indicate significant differences among all treatments at P < 0.05 according to the LSD tests. The results showed inoculations could increase the total contents of P, K, Mg and Fe in *P. americana*.


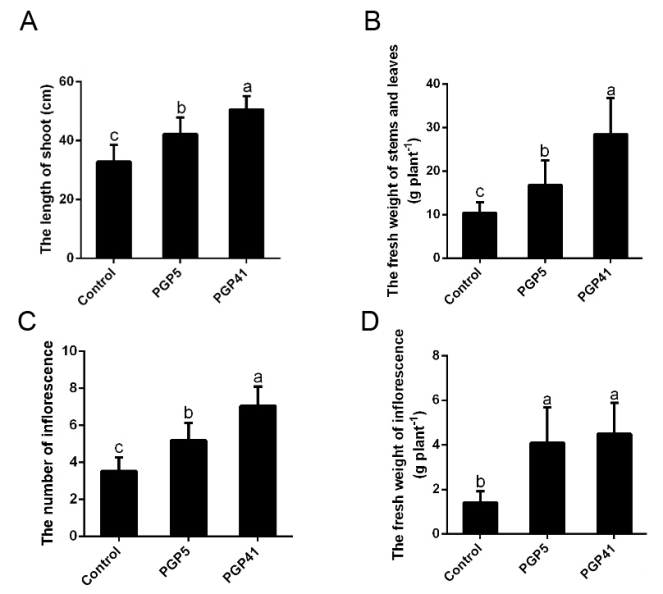


**Fig. S17.** Effects of inoculation of strain PGP5 or PGP41 on height (A), fresh weight of stems and leaves (B), number of inflorescences (C), and fresh weight of inflorescences (D) of *P. americana*. Values are means ± SD of three biological replicates and different lowercase letters indicate significant differences among all treatments at *P* < 0.05 according to the LSD tests. The data were got from field experiments conducted in Zhuzhou, Hunan Province, China (27°34’N, 113°12’E). The *P. americana* were sown in a 0.33 km^2^ plot, with row spacing of 30 cm. Each plant was inoculated once a month with 50 mL of distilled water (control) or microbial agents of PGP5 and PGP41 (PGP5 and PGP6). At 90 days after inoculation, the plants were sampled for measuring the plant growth physiological parameters.


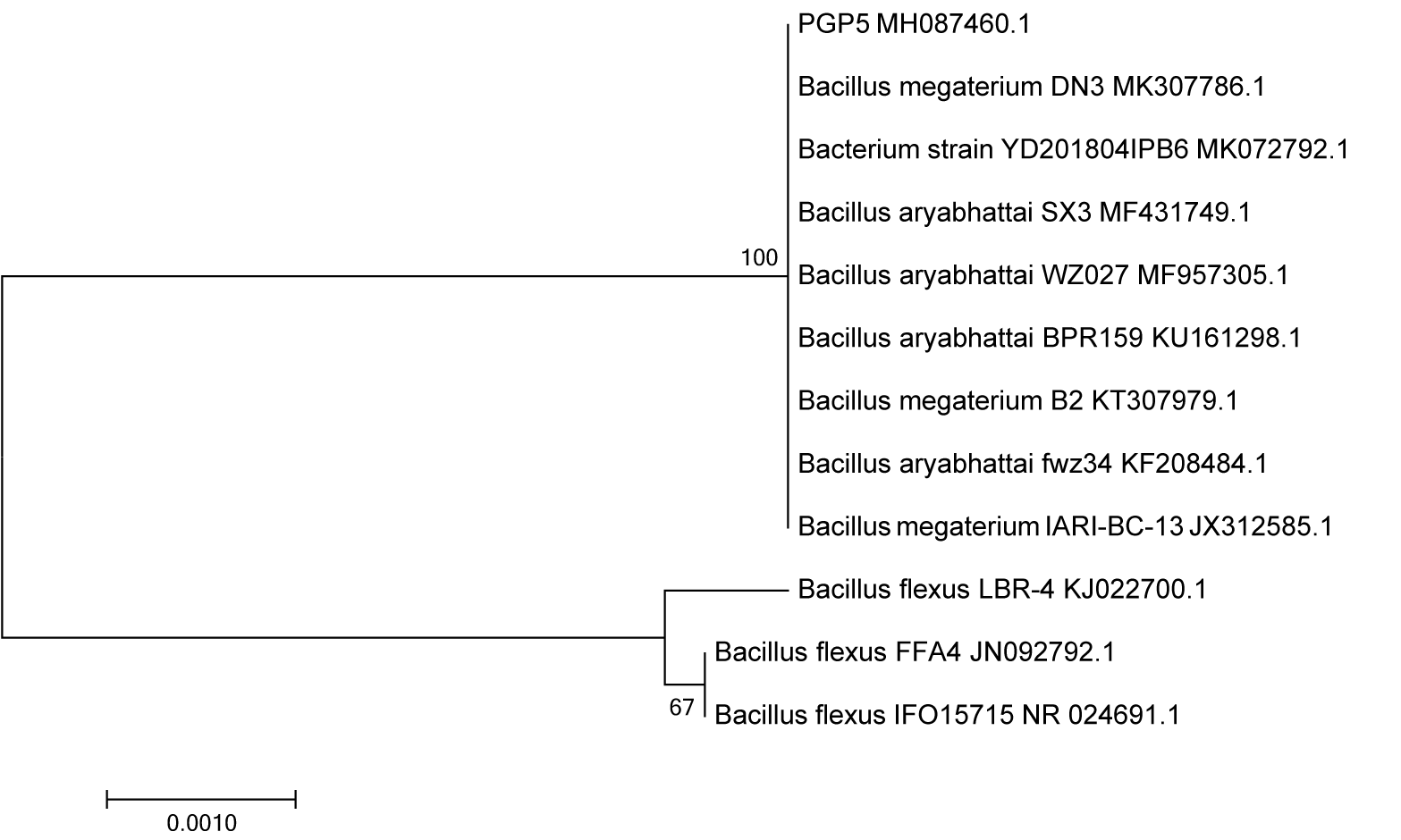


**Fig. S18**. Neighbor-joining tree based on 16S rRNA gene sequences showing the position of strain PGP41 within the genus Bacillus. Bootstrap values are shown at nodes as percentages of 1000 replicates.


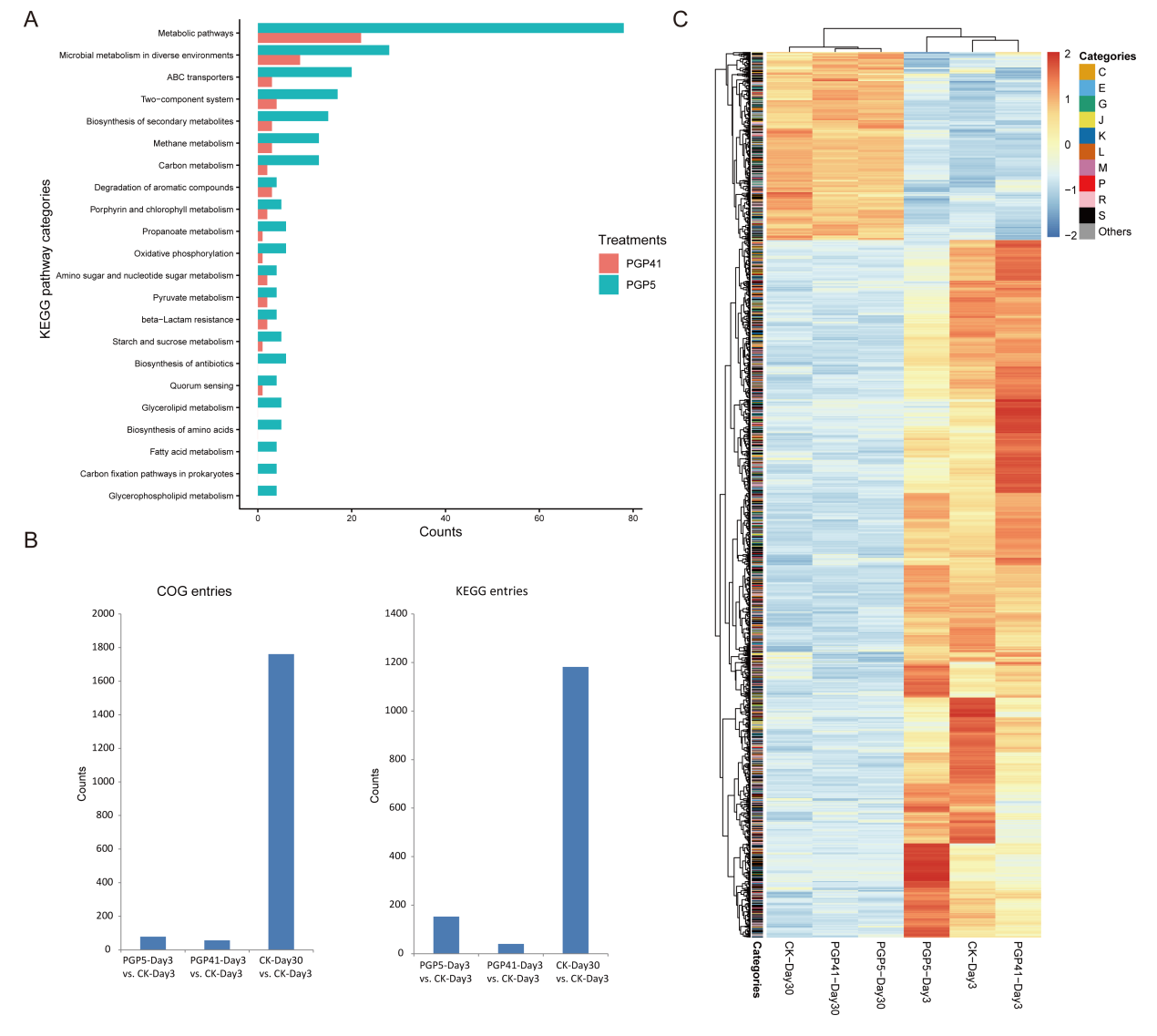


**Fig. S19**. Variation in the rhizosphere microbiome between Day 3 and Day 30 at the functional level. (A) Abundance of significantly changed KEGG categories in the PGP41- and PGP5-inoculated microbiomes compared to CK at Day 3. (B) Counts of significantly changed COG and KEGG categories in comparisons of PGP5-Day 3 vs. CK-Day 3, PGP41-Day 3 vs. CK-Day 3, and CK-Day 30 vs. CK-Day 3. (C) Heatmaps showing the average abundances of significantly changed COG categories in comparison of CK-Day 30 vs. CK-Day 3. COG categories are abbreviated as follows: C, energy production and conversion; E, amino acid transport and metabolism; G, carbohydrate transport and metabolism; J, translation, ribosomal structure and biogenesis; K, transcription; L, replication, recombination, and repair; M, cell wall/membrane/envelope biogenesis; P, inorganic ion transport and metabolism; Q, secondary metabolites biosynthesis, transport, and catabolism; R, general function prediction only; and S, function unknown.

**Supplementary Tables**

**Table S1.** GO enrichment analyses of PGP5 DEGs. The green cells refer to GO entries shared between Day 30 and Day 3.

| Id | Description | | p_fdr |
| --- | --- | --- | --- |
| PGP5-Day 30 | | | |
| GO:0005576 | extracellular region | | 0 |
| GO:0016798 | hydrolase activity, acting on glycosyl bonds | | 0 |
| GO:0004553 | hydrolase activity, hydrolyzing O-glycosyl compounds | | 0 |
| GO:0055114 | oxidation-reduction process | | 0 |
| GO:0016491 | oxidoreductase activity | | 0 |
| GO:0003824 | catalytic activity | | 0 |
| GO:0008150 | biological_process | | 0 |
| GO:0003674 | molecular_function | | 0 |
| GO:0043167 | ion binding | | 0 |
| GO:0008152 | metabolic process | | 0 |
| GO:0005975 | carbohydrate metabolic process | | 0 |
| GO:0046872 | metal ion binding | | 0 |
| GO:0016787 | hydrolase activity | | 0 |
| GO:0005488 | binding | | 0 |
| GO:0030145 | manganese ion binding | | 0 |
| GO:0046906 | tetrapyrrole binding | | 0 |
| GO:0016762 | xyloglucan:xyloglucosyl transferase activity | | 0 |
| GO:0020037 | heme binding | | 0 |
| GO:0005215 | transporter activity | | 0.004 |
| GO:0045735 | nutrient reservoir activity | | 0.006 |
| GO:0042744 | hydrogen peroxide catabolic process | | 0.008 |
| GO:0044262 | cellular carbohydrate metabolic process | | 0.008 |
| GO:0048037 | cofactor binding | | 0.008 |
| GO:0071555 | cell wall organization | | 0.008 |
| GO:0051187 | cofactor catabolic process | | 0.008 |
| GO:0005618 | cell wall | | 0.008 |
| GO:0045229 | external encapsulating structure organization | | 0.008 |
| GO:0005575 | cellular_component | | 0.008 |
| **PGP5-Day 3** | | | |
| GO:0006414 | | translational elongation | 0 |
| GO:0003924 | | GTPase activity | 0 |
| GO:0005576 | | extracellular region | 0 |
| GO:0004553 | | hydrolase activity, hydrolyzing O-glycosyl compounds | 0 |
| GO:0016798 | | hydrolase activity, acting on glycosyl bonds | 0 |
| GO:0005198 | | structural molecule activity | 0 |
| GO:0006412 | | translation | 0 |
| GO:0044271 | | cellular nitrogen compound biosynthetic process | 0 |
| GO:0003735 | | structural constituent of ribosome | 0 |
| GO:0009059 | | macromolecule biosynthetic process | 0 |
| GO:0034645 | | cellular macromolecule biosynthetic process | 0 |
| GO:0006518 | | peptide metabolic process | 0 |
| GO:0044249 | | cellular biosynthetic process | 0 |
| GO:1901576 | | organic substance biosynthetic process | 0 |
| GO:0009058 | | biosynthetic process | 0 |
| GO:0030529 | | intracellular ribonucleoprotein complex | 0 |
| GO:0043170 | | macromolecule metabolic process | 0 |
| GO:0016787 | | hydrolase activity | 0 |
| GO:0001883 | | purine nucleoside binding | 0 |
| GO:0043604 | | amide biosynthetic process | 0 |
| GO:0043603 | | cellular amide metabolic process | 0 |
| GO:0005525 | | GTP binding | 0 |
| GO:0071704 | | organic substance metabolic process | 0 |
| GO:0008152 | | metabolic process | 0 |
| GO:0003824 | | catalytic activity | 0 |
| GO:0019001 | | guanyl nucleotide binding | 0 |
| GO:0008150 | | biological_process | 0 |
| GO:0005575 | | cellular_component | 0 |
| GO:0003674 | | molecular_function | 0 |
| GO:0032549 | | ribonucleoside binding | 0 |
| GO:0044260 | | cellular macromolecule metabolic process | 0 |
| GO:0009987 | | cellular process | 0 |
| GO:0044237 | | cellular metabolic process | 0 |
| GO:0044464 | | cell part | 0 |
| GO:1901566 | | organonitrogen compound biosynthetic process | 0 |
| GO:0016762 | | xyloglucan:xyloglucosyl transferase activity | 0 |
| GO:0008135 | | translation factor activity, RNA binding | 0 |
| GO:0044036 | | cell wall macromolecule metabolic process | 0 |
| GO:0044238 | | primary metabolic process | 0 |
| GO:0010410 | | hemicellulose metabolic process | 0 |
| GO:0010411 | | xyloglucan metabolic process | 0 |
| GO:0010383 | | cell wall polysaccharide metabolic process | 0 |
| GO:0045229 | | external encapsulating structure organization | 0 |
| GO:0071555 | | cell wall organization | 0 |
| GO:0005975 | | carbohydrate metabolic process | 0 |
| GO:0005840 | | ribosome | 0 |
| GO:0042546 | | cell wall biogenesis | 0 |
| GO:0043167 | | ion binding | 0 |
| GO:0020037 | | heme binding | 0 |
| GO:0046906 | | tetrapyrrole binding | 0 |
| GO:0071554 | | cell wall organization or biogenesis | 0 |
| GO:0042744 | | hydrogen peroxide catabolic process | 0.002 |
| GO:0044391 | | ribosomal subunit | 0.002 |
| GO:0042743 | | hydrogen peroxide metabolic process | 0.002 |
| GO:0042737 | | drug catabolic process | 0.002 |
| GO:0005488 | | binding | 0.004 |
| GO:0044424 | | intracellular part | 0.004 |
| GO:0051187 | | cofactor catabolic process | 0.004 |
| GO:0055114 | | oxidation-reduction process | 0.004 |
| GO:0000027 | | ribosomal large subunit assembly | 0.004 |
| GO:0005215 | | transporter activity | 0.004 |
| GO:0003723 | | RNA binding | 0.004 |
| GO:0019538 | | protein metabolic process | 0.004 |
| GO:0015934 | | large ribosomal subunit | 0.008 |
| GO:0044444 | | cytoplasmic part | 0.008 |
| GO:0044262 | | cellular carbohydrate metabolic process | 0.008 |
| GO:0005618 | | cell wall | 0.008 |

**Table S2**. GO enrichment analyses of PGP41 DEGs. The green cells refer to GO entries shared between Day 30 and Day 3.

| Id | Description | p_fdr |
| --- | --- | --- |
| **PGP41-Day 30** | | |
| GO:0006414 | translational elongation | 0 |
| GO:0003924 | GTPase activity | 0 |
| GO:0005576 | extracellular region | 0 |
| GO:0004553 | hydrolase activity, hydrolyzing O-glycosyl compounds | 0 |
| GO:0016798 | hydrolase activity, acting on glycosyl bonds | 0 |
| GO:0005198 | structural molecule activity | 0 |
| GO:0006412 | translation | 0 |
| GO:0044271 | cellular nitrogen compound biosynthetic process | 0 |
| GO:0003735 | structural constituent of ribosome | 0 |
| GO:0009059 | macromolecule biosynthetic process | 0 |
| GO:0034645 | cellular macromolecule biosynthetic process | 0 |
| GO:0006518 | peptide metabolic process | 0 |
| GO:0044249 | cellular biosynthetic process | 0 |
| GO:1901576 | organic substance biosynthetic process | 0 |
| GO:0009058 | biosynthetic process | 0 |
| GO:0030529 | intracellular ribonucleoprotein complex | 0 |
| GO:0043170 | macromolecule metabolic process | 0 |
| GO:0016787 | hydrolase activity | 0 |
| GO:0001883 | purine nucleoside binding | 0 |
| GO:0043604 | amide biosynthetic process | 0 |
| GO:0043603 | cellular amide metabolic process | 0 |
| GO:0005525 | GTP binding | 0 |
| GO:0071704 | organic substance metabolic process | 0 |
| GO:0008152 | metabolic process | 0 |
| GO:0003824 | catalytic activity | 0 |
| GO:0019001 | guanyl nucleotide binding | 0 |
| GO:0008150 | biological_process | 0 |
| GO:0005575 | cellular_component | 0 |
| GO:0003674 | molecular_function | 0 |
| GO:0032549 | ribonucleoside binding | 0 |
| GO:0044260 | cellular macromolecule metabolic process | 0 |
| GO:0009987 | cellular process | 0 |
| GO:0044237 | cellular metabolic process | 0 |
| GO:0044464 | cell part | 0 |
| GO:1901566 | organonitrogen compound biosynthetic process | 0 |
| GO:0016762 | xyloglucan:xyloglucosyl transferase activity | 0 |
| GO:0008135 | translation factor activity, RNA binding | 0 |
| GO:0044036 | cell wall macromolecule metabolic process | 0 |
| GO:0044238 | primary metabolic process | 0 |
| GO:0010410 | hemicellulose metabolic process | 0 |
| GO:0010411 | xyloglucan metabolic process | 0 |
| GO:0010383 | cell wall polysaccharide metabolic process | 0 |
| GO:0045229 | external encapsulating structure organization | 0 |
| GO:0071555 | cell wall organization | 0 |
| GO:0005975 | carbohydrate metabolic process | 0 |
| GO:0005840 | ribosome | 0 |
| GO:0042546 | cell wall biogenesis | 0 |
| GO:0043167 | ion binding | 0 |
| GO:0020037 | heme binding | 0 |
| GO:0046906 | tetrapyrrole binding | 0 |
| GO:0071554 | cell wall organization or biogenesis | 0 |
| GO:0042744 | hydrogen peroxide catabolic process | 0.002 |
| GO:0044391 | ribosomal subunit | 0.002 |
| GO:0042743 | hydrogen peroxide metabolic process | 0.002 |
| GO:0042737 | drug catabolic process | 0.002 |
| GO:0005488 | binding | 0.004 |
| GO:0044424 | intracellular part | 0.004 |
| GO:0051187 | cofactor catabolic process | 0.004 |
| GO:0055114 | oxidation-reduction process | 0.004 |
| GO:0000027 | ribosomal large subunit assembly | 0.004 |
| GO:0005215 | transporter activity | 0.004 |
| GO:0003723 | RNA binding | 0.004 |
| GO:0019538 | protein metabolic process | 0.004 |
| GO:0015934 | large ribosomal subunit | 0.008 |
| GO:0044444 | cytoplasmic part | 0.008 |
| GO:0044262 | cellular carbohydrate metabolic process | 0.008 |
| GO:0005618 | cell wall | 0.008 |
| **PGP41-Day 3** | | |
| GO:0009664 | plant-type cell wall organization | 0 |
| GO:0051187 | cofactor catabolic process | 0 |
| GO:0004601 | peroxidase activity | 0 |
| GO:0016684 | oxidoreductase activity, acting on peroxide as acceptor | 0 |
| GO:0071669 | plant-type cell wall organization or biogenesis | 0 |
| GO:0042744 | hydrogen peroxide catabolic process | 0 |
| GO:0016831 | carboxy-lyase activity | 0 |
| GO:0072593 | reactive oxygen species metabolic process | 0 |
| GO:0099513 | polymeric cytoskeletal fiber | 0 |
| GO:0016209 | antioxidant activity | 0 |
| GO:0071555 | cell wall organization | 0 |
| GO:0005618 | cell wall | 0 |
| GO:0098869 | cellular oxidant detoxification | 0 |
| GO:0099512 | supramolecular fiber | 0 |
| GO:0006979 | response to oxidative stress | 0 |
| GO:0042743 | hydrogen peroxide metabolic process | 0 |
| GO:1990748 | cellular detoxification | 0 |
| GO:0098754 | detoxification | 0 |
| GO:0005874 | microtubule | 0 |
| GO:0071554 | cell wall organization or biogenesis | 0 |
| GO:0022625 | cytosolic large ribosomal subunit | 0 |
| GO:0045229 | external encapsulating structure organization | 0 |
| GO:0020037 | heme binding | 0 |
| GO:0007017 | microtubule-based process | 0 |
| GO:0046906 | tetrapyrrole binding | 0 |
| GO:0015934 | large ribosomal subunit | 0 |
| GO:0005576 | extracellular region | 0 |
| GO:0015935 | small ribosomal subunit | 0 |
| GO:0017144 | drug metabolic process | 0 |
| GO:0005200 | structural constituent of cytoskeleton | 0 |
| GO:0016798 | hydrolase activity, acting on glycosyl bonds | 0 |
| GO:0044391 | ribosomal subunit | 0 |
| GO:0004553 | hydrolase activity, hydrolyzing O-glycosyl compounds | 0 |
| GO:0044445 | cytosolic part | 0 |
| GO:0006468 | protein phosphorylation | 0 |
| GO:0004672 | protein kinase activity | 0 |
| GO:0043604 | amide biosynthetic process | 0 |
| GO:0005215 | transporter activity | 0 |
| GO:0022857 | transmembrane transporter activity | 0 |
| GO:0006518 | peptide metabolic process | 0 |
| GO:0006412 | translation | 0 |
| GO:0005975 | carbohydrate metabolic process | 0 |
| GO:0016301 | kinase activity | 0 |
| GO:0005198 | structural molecule activity | 0 |
| GO:0030529 | intracellular ribonucleoprotein complex | 0 |
| GO:0055085 | transmembrane transport | 0 |
| GO:0003735 | structural constituent of ribosome | 0 |
| GO:0016773 | phosphotransferase activity, alcohol group as acceptor | 0 |
| GO:0043043 | peptide biosynthetic process | 0 |
| GO:0043603 | cellular amide metabolic process | 0 |
| GO:0016310 | phosphorylation | 0 |
| GO:0006464 | cellular protein modification process | 0 |
| GO:0050896 | response to stimulus | 0 |
| GO:0055114 | oxidation-reduction process | 0 |
| GO:0034645 | cellular macromolecule biosynthetic process | 0 |
| GO:0016020 | membrane | 0 |
| GO:1901566 | organonitrogen compound biosynthetic process | 0 |
| GO:0016491 | oxidoreductase activity | 0 |
| GO:0044271 | cellular nitrogen compound biosynthetic process | 0 |
| GO:0006793 | phosphorus metabolic process | 0 |
| GO:0009059 | macromolecule biosynthetic process | 0 |
| GO:0140096 | catalytic activity, acting on a protein | 0 |
| GO:0032559 | adenyl ribonucleotide binding | 0 |
| GO:0005524 | ATP binding | 0 |
| GO:0030554 | adenyl nucleotide binding | 0 |
| GO:0017076 | purine nucleotide binding | 0 |
| GO:0006796 | phosphate-containing compound metabolic process | 0 |
| GO:1901576 | organic substance biosynthetic process | 0 |
| GO:0046872 | metal ion binding | 0 |
| GO:0043169 | cation binding | 0 |
| GO:0044267 | cellular protein metabolic process | 0 |
| GO:0008144 | drug binding | 0 |
| GO:0009058 | biosynthetic process | 0 |
| GO:0019538 | protein metabolic process | 0 |
| GO:0044249 | cellular biosynthetic process | 0 |
| GO:0035639 | purine ribonucleoside triphosphate binding | 0 |
| GO:0097367 | carbohydrate derivative binding | 0 |
| GO:0016787 | hydrolase activity | 0 |
| GO:0016772 | transferase activity, transferring phosphorus-containing groups | 0 |
| GO:0043168 | anion binding | 0 |
| GO:0016740 | transferase activity | 0 |
| GO:0032553 | ribonucleotide binding | 0 |
| GO:0032555 | purine ribonucleotide binding | 0 |
| GO:0016021 | integral component of membrane | 0 |
| GO:0044425 | membrane part | 0 |
| GO:1901564 | organonitrogen compound metabolic process | 0 |
| GO:0031224 | intrinsic component of membrane | 0 |
| GO:0044260 | cellular macromolecule metabolic process | 0 |
| GO:0043170 | macromolecule metabolic process | 0 |
| GO:0006807 | nitrogen compound metabolic process | 0 |
| GO:0043167 | ion binding | 0 |
| GO:1901363 | heterocyclic compound binding | 0 |
| GO:0044237 | cellular metabolic process | 0 |
| GO:0044464 | cell part | 0 |
| GO:0043412 | macromolecule modification | 0 |
| GO:0071704 | organic substance metabolic process | 0 |
| GO:0044238 | primary metabolic process | 0 |
| GO:1901265 | nucleoside phosphate binding | 0 |
| GO:0044424 | intracellular part | 0 |
| GO:0036094 | small molecule binding | 0 |
| GO:0009987 | cellular process | 0 |
| GO:0003824 | catalytic activity | 0 |
| GO:0005488 | binding | 0 |
| GO:0008152 | metabolic process | 0 |
| GO:0009056 | catabolic process | 0 |
| GO:0005575 | cellular_component | 0 |
| GO:0008150 | biological_process | 0 |
| GO:0006810 | transport | 0 |
| GO:0003674 | molecular_function | 0 |
| GO:0051234 | establishment of localization | 0 |
| GO:0051179 | localization | 0 |
| GO:0006950 | response to stress | 0 |
| GO:0008964 | phosphoenolpyruvate carboxylase activity | 0 |
| GO:0048037 | cofactor binding | 0 |
| GO:0016830 | carbon-carbon lyase activity | 0 |
| GO:0004674 | protein serine/threonine kinase activity | 0.002 |
| GO:0044444 | cytoplasmic part | 0.002 |
| GO:0016757 | transferase activity, transferring glycosyl groups | 0.002 |
| GO:0044430 | cytoskeletal part | 0.002 |
| GO:0015977 | carbon fixation | 0.002 |
| GO:0003924 | GTPase activity | 0.002 |
| GO:0005886 | plasma membrane | 0.002 |
| GO:0044248 | cellular catabolic process | 0.002 |
| GO:0022627 | cytosolic small ribosomal subunit | 0.002 |
| GO:0006811 | ion transport | 0.002 |
| GO:0044446 | intracellular organelle part | 0.002 |
| GO:0055082 | cellular chemical homeostasis | 0.002 |
| GO:0072351 | tricarboxylic acid biosynthetic process | 0.002 |
| GO:0031226 | intrinsic component of plasma membrane | 0.002 |
| GO:0016628 | oxidoreductase activity, acting on the CH-CH group of donors, NAD or NADP as acceptor | 0.002 |
| GO:0015291 | secondary active transmembrane transporter activity | 0.002 |
| GO:0032991 | macromolecular complex | 0.002 |
| GO:0044262 | cellular carbohydrate metabolic process | 0.002 |
| GO:0009506 | plasmodesma | 0.002 |
| GO:0015791 | polyol transport | 0.002 |
| GO:0006629 | lipid metabolic process | 0.002 |
| GO:0044459 | plasma membrane part | 0.002 |
| GO:0005976 | polysaccharide metabolic process | 0.004 |
| GO:0032440 | 2-alkenal reductase [NAD(P)] activity | 0.004 |
| GO:0051213 | dioxygenase activity | 0.004 |
| GO:0051186 | cofactor metabolic process | 0.006 |
| GO:0005509 | calcium ion binding | 0.006 |
| GO:0006820 | anion transport | 0.006 |
| GO:0015631 | tubulin binding | 0.006 |
| GO:0004190 | aspartic-type endopeptidase activity | 0.006 |
| GO:0048878 | chemical homeostasis | 0.006 |

**Table S3**. Primers used in this study.

|  | Sense (5'-3') | Anti-sense (5'-3') |
| --- | --- | --- |
| **Primers of 16s RNA gene** | | |
| universal | AGAGTTTGATCCTGGCTCAG | GGTTACCTTGTTACGACTT |
| PGP5-spedific 1 | TTGACCTCGCGGTCTTGCA | TGATGAAGGCTTTCGGGTCG |
| PGP5-spedific 2 | CGCTCTATCTCTAGAGTTGT | CTTCTCCTTCATGGGAGATGAT |
| PGP5-spedific 3 | CGCTCTATCTCTAGAGTTGT | TGATGAAGGCTTTCGGGTCG |
| PGP41-spedific 1 | TGGTGAGGTAATGGCTTAC | TGTAAACCGACCGCAAGC |
| PGP41-spedific 2 | TGGTGAGGTAATGGCTTAC | TCTCCAGGCGTTTCCGGTT |
| **Primers of housekeeping genes** | | |
| actin | AGTACCCGATTGAGCATGGTATTG | TGATCTGAGTCATCTTCTCCCTGT |
| tublin | TCGTGCCGTGTTTGTAGATCTG | CCTTTCCGCTAATCAGCTGCT |
| **Primers of genes involved in maintaining DNA methylation** | | |
| DN117084 | CTCTTCCTGGAGATGTGG | TTCGTGCTTGGTAGTTCA |
| DN50796 | TGCTGACTGGCGAGAT | CTTGCCCATTGGTTGT |
| DN68042 | AGGAATGCACCCGTAG | CCCAACCCAGTCGTAT |
| DN81080 | TGGAATGTCAAGGGAA | CAGGAGGTTGGATAGAAT |
| DN76720 | ACTTCATCCAAGCCATTT | GCAACAGCATTCCCTAC |
| DN77575 | TGTCAAGGCATAAGTGG | GCATTAGCGAACCTCA |
| **Primers of DEG-DMRs overlapped genes** | | |
| DN105594 | GCACCCACCAACCGTATTTCTTC | GCATATCCTCCTTCCAGCACCAA |
| DN117054 | CCTCCCTTGGTACTGTTTGAC | GAGAATCTACGGCGAACTTGA |
| DN20544 | AGGATGAGTAGGATATGTGAAGGT | GCTGGTCAGTATCCGATGTTG |
| DN20966 | GTGGTGATGTTGTTGATGGAGATG | GCCAGCCAAGGTAGGATCATC |
| DN29461 | CGTGGGTAGTCATATCAGTGTAGC | AACTCCGCCTGTGAAGCCTTA |
| DN41808 | ACAACTCACCTGCCGAATG | CGCTTCTCCTTAGTATTTGCTAC |
| DN6511 | CGACCAGGATTATTTGTTCGTTGA | GAGCACAAGAGGTGAGCAGTT |
| DN71200 | CACTAATAGCAGCAATGTCCTT | AGTCTTGATTATGGTCGGAGAA |
| DN72842 | TCTGGAAGCGACCAAT | TCACCAACGGGATGTA |
| DN76868 | GCATGGCCTTCACCTTGT | GATGAGCATCATTAACCGAAGT |
| DN79851 | GGTCTATTCGGTTCGGTTCTAAT | GGGAAGAGGAAGGACGGAAA |
| DN79927 | GGCAACAGCATAAGATGGTAGTC | TGGCAGTGAGCAAGAGGAAG |
| DN81311 | TAGACCAAGGAACCGTGAGAAGT | CAGGAGATTGCTGGAGATGAAGG |
| DN82480 | CTGAGATTGGTAGGACTGTGTAGC | CTGGAAGGCAAGAACTTAGAAAGC |
| DN90023 | AGACCGCCGTTGCTGTT | CCTCTCGCTCTCTTTCTCTCT |
| **Primers of 16s RNA gene for qPCR** | | |
| PGP5-16s-qPCR | CATCGTTGCCTTGGTGAG | CTTCTCCTTCATGGGAGATGAT |
| PGP41-16s-qPCR | GGAGCGAACAGGATTAGATACC | TCTCCAGGCGTTTCCGGTT |
| **FISH probes** | | |
| PGP5 | CTGGAGACGCAACGTGAATGCCAAGCT | |
| PGP41 | CACCTGTCTCCAGGCGTTTCCGGTTC | |
